# Identification of a Negative Allosteric Site on Human α4β2 and α3β4 Neuronal Nicotinic Acetylcholine Receptors

**DOI:** 10.1371/journal.pone.0024949

**Published:** 2011-09-15

**Authors:** Ryan E. Pavlovicz, Brandon J. Henderson, Andrew B. Bonnell, R. Thomas Boyd, Dennis B. McKay, Chenglong Li

**Affiliations:** 1 Biophysics Program, Ohio State University, Columbus, Ohio, United States of America; 2 Division of Pharmacology, College of Pharmacy, Ohio State University, Columbus, Ohio, United States of America; 3 Department of Neuroscience, Ohio State University, Columbus, Ohio, United States of America; 4 Division of Medicinal Chemistry and Pharmacognosy, College of Pharmacy, Ohio State University, Columbus, Ohio, United States of America; Massachusetts Institute of Technology, United States of America

## Abstract

Acetylcholine-based neurotransmission is regulated by cationic, ligand-gated ion channels called nicotinic acetylcholine receptors (nAChRs). These receptors have been linked to numerous neurological diseases and disorders such as Alzheimer's disease, Parkinson's disease, and nicotine addiction. Recently, a class of compounds has been discovered that antagonize nAChR function in an allosteric fashion. Models of human α4β2 and α3β4 nicotinic acetylcholine receptor (nAChR) extracellular domains have been developed to computationally explore the binding of these compounds, including the dynamics and free energy changes associated with ligand binding. Through a blind docking study to multiple receptor conformations, the models were used to determine a putative binding mode for the negative allosteric modulators. This mode, in close proximity to the agonist binding site, is presented in addition to a hypothetical mode of antagonism that involves obstruction of C loop closure. Molecular dynamics simulations and MM-PBSA free energy of binding calculations were used as computational validation of the predicted binding mode, while functional assays on wild-type and mutated receptors provided experimental support. Based on the proposed binding mode, two residues on the β2 subunit were independently mutated to the corresponding residues found on the β4 subunit. The T58K mutation resulted in an eight-fold decrease in the potency of KAB-18, a compound that exhibits preferential antagonism for human α4β2 over α3β4 nAChRs, while the F118L mutation resulted in a loss of inhibitory activity for KAB-18 at concentrations up to 100 µM. These results demonstrate the selectivity of KAB-18 for human α4β2 nAChRs and validate the methods used for identifying the nAChR modulator binding site. Exploitation of this site may lead to the development of more potent and subtype-selective nAChR antagonists which may be used in the treatment of a number of neurological diseases and disorders.

## Introduction

Nicotinic acetylcholine receptors (nAChRs) are ligand-gated, cation channels found throughout the central and peripheral nervous systems [1], [2], [3]. Physiologically, neuronal nAChRs are complex, participating in many neurological processes including cognition [4], pain sensation [5], and nicotine reward/addiction mechanisms [6], [7]. In addition to nicotine addiction, these receptors have been linked to numerous neurological diseases and disorders including Parkinson's disease [8], Alzheimer's disease [8], schizophrenia [9], epilepsy [10], and lung cancer [11], making them important therapeutic targets.

Pentameric in assembly, these plasma membrane channels may be classified as either muscle- or neuronal-type receptors based on their subunit composition. There are numerous subtypes of neuronal nAChRs, with α2-α10 and β2-β4 subunits arranging in either homo- or heteropentameric assemblies. The heteromeric receptors contain both α and β subunits, with a general stoichiometry of 2α:3β [12], [13], [14], although there is also evidence for (α4)_3_(β2)_2_ nAChRs [15], [16]. The homomeric receptors are solely comprised of α subunits and have five agonist binding sites, while the heteromeric receptors have two agonist binding sites. For heteromeric receptors, agonist binding occurs at α(+)/β(-) interfaces, where the (+) notation implies the contribution of a principle ligand-binding feature called the C loop to the binding interface and the (-) notation refers to the complementary subunit surface that completes the binding site.

Because the composition and distribution of nAChRs throughout the nervous system are so varied, it is difficult to study the roles of the various nAChR subtypes in neuronal signaling pathways. In order to deduce these functional roles, there is a need for nAChR antagonists that selectively target specific receptor subtypes. Agonist binding at the α/β interface involves interactions with a group of five aromatic residues often called the “aromatic nest”. Since these agonist-binding residues are conserved in all nAChR subunits, it is difficult to design selective nAChR molecules that target the agonist binding site. Therefore, targeting allosteric binding sites may be a more viable strategy in the development of subtype-selective nAChR antagonists. Due to the emergence of crystallographic structures that aid in the modeling of various subtype assemblies, the ability to implement rational, structure-based drug design techniques to the development of subtype-selective nAChR antagonists is becoming an increasingly attainable goal.

**Figure 1:**
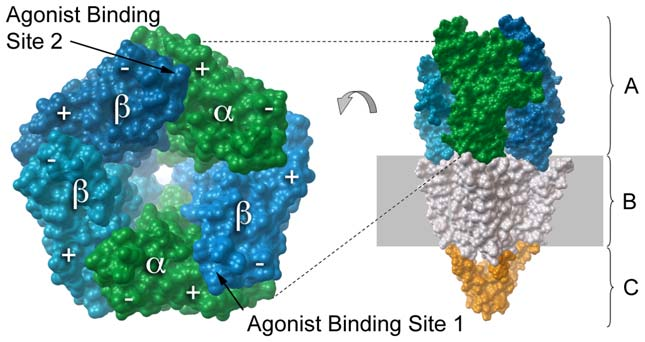
Schematic of neuronal nAChR structure. The proportions of the extracellular domain (A), transmembrane domain (B), and intracellular domain (C) are illustrated on the right while the modeled subunit stoichiometry and configuration for heteromeric neuronal nAChRs is illustrated on the left, including labels for the (+) and (−) side of each subunit and the location of each of the two agonist binding sites.

The general nAChR structure (Fig. 1) is known from electron microscopy (EM) data of the *Torpedo marmorata* muscle-type receptor [17]. Structural comparison between the muscle-type nAChR and acetylcholine binding protein (AChBP), a soluble pentamer found in molluskan species, revealed that AChBP is a structural homologue of the extracellular domain (ECD) of nAChRs [18]. AChBP structures have been reported for three different molluskan species [18], [19], [20]. The most recent nAChR-related structure is that of the α1 extracellular domain of the mouse nAChR [21]. These structures aid three-dimensional modeling of nAChRs, with previous studies addressing topics such as gating dynamics [22], [23], agonist binding [24], [25], [26], agonist selectivity [27], [28], and allosteric modulator binding [29]. More recently, some studies have eschewed nAChR modeling all together, using AChBP structures directly in virtual screening attempts to identify novel nAChR ligands [30], [31].

**Figure 2:**
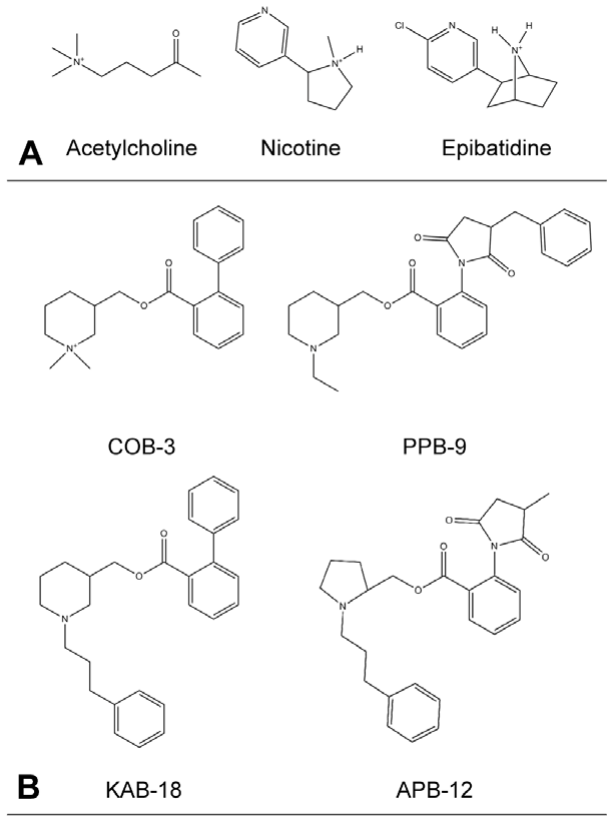
Compounds used in hα3β4 and hα4β2 nAChR ECD blind docking. **A**. Docked agonists included acetylcholine, nicotine, and epibatidine. **B**. Docked antagonists included COB-3, PPB-9, APB-12, and KAB-18.

Since most nAChR-related experimental structures support modeling of the ECD, and this receptor domain is known to bind a number of ligands with varied pharmacological effects, the ECD is the focus of this computational study. In this paper, we model human (α4)_2_(β2)_3_ (hα4β2) and human (α3)_2_(β4)_3_ (hα3β4) nAChR extracellular domains based on multiple crystallographic templates and utilize empirical experimental data [32] to validate the models. Human α4β2 nAChRs are the major nAChR subtype present in brain and are a target for development of drugs for smoking cessation and other brain cholinergic disorders while human α3β4 nAChRs are the major ganglionic nAChRs and often mediate undesirable off-target effects of smoking cessation drugs. After validating the feasibility of docking to the models with a set of agonists with established binding modes, we probed the models for a binding site of a unique set of antagonists (Fig. 2B), recently described as negative allosteric modulators (NAMs), that act via non-competitive mechanisms to inhibit activation of nAChRs [33], [34]. Some of these compounds, including a molecule called KAB-18, exhibit preferential inhibition of hα4β2 nAChRs when compared to hα3β4 nAChRs, however potency of these molecules is limited to the low µM range (10 µM for KAB-18). Therefore, studying how these compounds specifically interact with the receptor can allow us to design more potent drugs that inhibit nAChRs of specific subunit compositions. Molecular dynamics (MD) simulations and free energy calculations of the antagonist KAB-18 bound to both models are used to support the validity of the proposed binding mode. Additionally, functional data involving mutated nAChRs are presented to further support the computationally determined binding site.

## Materials and Methods

### Modeling

The extracellular domains of two different human nAChR subtypes were modeled for use in this computational study: (α4)_2_(β2)_3_ and (α3)_2_(β4)_3_. Hereafter, all references to nAChR models refer to the extracellular domain only. Four different crystallographic templates were used in the homology modeling process: three different molluskan species of AChBP (PDB IDs: 1UW6 [35], 2BYR [36], and 2BJ0 [19]) as well as the mouse α1 ECD monomer (PDB ID: 2QC1 [21]). An artificial α1 pentamer was created by superimposing the monomer over an AChBP structure five separate times.

The alignment of the four template structures to the target sequences was largely performed manually, although cues were taken from PSIPRED [37] and PHD [38] secondary structure predictions (See Fig. S1, Table S1, and Table S2 for alignment and homology information). Alignment algorithms were not used due to the low identity between the target (nAChR) and template (AChBP) sequences. The insertions and/or deletions in loops regions coupled with the low sequence identity resulted in poor alignments, therefore a manual alignment based on conserving structural features was deemed appropriate. Following alignment, three-dimensional models were built with MODELLER9v1 [39] in an iterative fashion, with 200 models being built in each iteration. Since the model assessment methods used in MODELLER were exclusively calibrated with single-chain proteins, they are not suitable for selecting top structures among the pentameric nAChR models. To more accurately select a top structure, each model was scored with a molecular mechanics – Poisson Boltzmann surface area (MM-PBSA) approach. Each model was solvated in a TIP3P water box, energy minimized, stripped of its waters, then scored with a Poisson Boltzmann approach in the Amber 9 suite of programs [40].

Initially, a rat α3β4 (rα3β4) nAChR model was created in six modeling iterations, where each successive iteration added additional symmetry, distance, and secondary structural restraints as well as incorporating the best-ranking model from the previous iteration as a fifth template structure. Molecular dynamics with locally enhanced sampling (LES) [41] was applied to the top structure of the sixth modeling iteration to better sample the conformation of the A loop and its connection to the adjoining β5 strand of each subunit. The loop and strand regions treated by the locally enhanced sampling (LES) method correspond to residues 94-105 for the α subunits and 96-107 for the β subunits (see Fig. S1 for numbering scheme). In total, there were 55 LES residues for which five copies of each were made. This left the remaining 987 residues of the models to be treated classically. The structure with the lowest computed energy during the five-copy, 4 ns LES-MD simulation was selected as the final template for homology modeling. The same templates and restraints used to obtain the final rα3β4 nAChR model were also used to create the human α3β4 and α4β2 nAChR models. The rα3β4 nAChR was initially modeled to complement available experimental data [33], [34]. Ultimately, we modeled the hα3β4 and hα4β2 nAChR to reflect a switch in receptors used by our experimental collaborators [32].

### Conformational Sampling of the Receptor

An ensemble of receptor snapshots was collected from a molecular dynamics (MD) trajectory to account for protein flexibility during docking. Constant volume and temperature MD simulations of the nAChR models used the SHAKE algorithm as implemented by Amber with a 2 fs time step. Prior to the production run, the model was solvated in a TIP3P water box with a 15 Å buffer around all edges of the protein. After solvation, the system was charge neutralized by the addition of Na+ counterions, and energy minimized by 500 steps of steepest descent minimization followed by 1500 steps of conjugate gradient minimization. The system was equilibrated by first increasing the temperature of the system from 0 K to 300 K over 200 ps in which all protein atoms were fixed with a 50 kcal/mol harmonic potential. This proved to be an important step, since it allowed the water molecules to fill in the gaps at the protein/water interface that were left vacant by the solvating algorithm in the LEaP module of Amber. If the waters were not first allowed to equilibrate around the protein, undesired side chain movements were observed that detrimentally effected agonist docking to the agonist binding sites. A final 200 ps of unrestrained MD completed the equilibration process. Production runs of 5 ns followed the equilibration. All simulations used a heat bath coupling constant of 2.0 ps and were performed at 1 atm with a pressure relaxation time of 2.0 ps. Nonbonded interaction calculations were cutoff at 8 Å, while the electrostatic energy was computed using the Particle Mesh Ewald method. The simulations were run using the sander code of Amber 9 with the ff99 force field. Snapshots were captured at 200 ps intervals along the production run trajectories to form a set of 26 receptor conformations that were used for docking.

### Blind Docking

Blind docking grids of size 90.00 Å × 90.00 Å × 56.25 Å with grid point spacing of 0.375 Å were constructed for each snapshot conformation with AutoGrid4. These grids were large enough to encompass the entire extracellular domain, only excluding the Cys-loop region, since docking results in this region are unrealistic due to the contact these loops make with the TM2-TM3 loops that are not part of these models.

Three different agonists with known experimental binding modes to AChBP were blindly docked to each of the receptor conformations collected from the MD trajectory. These compounds, illustrated in Fig. 2A, include acetylcholine, nicotine, and epibatidine. Agonist structural coordinates were taken from the PDB and processed by the LigPrep program of the Schrödinger suite to determine the ionization state of each compound at pH 7 ± 2. All agonists were determined to carry a positive charge within the pH range considered. All compounds were assigned Gasteiger charges and docked with the Lamarckian genetic algorithm (LGA) [42] in AutoDock4 [43] with the maximum number of freely rotating bonds per ligand. One hundred independent docking runs were completed for each ligand to each of the receptor conformations. A cutoff of 25,000,000 – 100,000,000 energy evaluations was used, depending on the number of rotatable bonds in the ligand, while all other docking parameters maintained the default setting.

Each of the 100 docking positions for each ligand at each receptor conformation were clustered by their centroid points with a 4 Å tolerance. The four most populous clusters of each ligand were then clustered against those from the other receptor conformations. This clustering of clusters was based on the receptor residues that came into contact with each cluster instead of the Cartesian coordinates attributed to the centroid-based clusters. This method allowed for the clusters from different time points to be compared to each other without having to worry about spatial drift or rotation of the receptor. A list of residues coming within 5 Å of each of the docked conformations for each centroid-based cluster was created with scripts utilizing functions available in the Chimera program [44]. Clusters with residue lists that shared a 65% intersection were considered to belong to the same docking position.

### Antagonist Docking to Ternary Complex

After the initial round of blind docking to the unbound nAChR models, the epibatidine docking with the smallest RMSD from the AChBP binding mode (as found in PDB ID: 2BYQ) was kept as part of each nAChR structure. Each agonist-bound system was then resampled via an MD simulation similar to the protocol described above. A second epibatidine molecule was then docked to the models using the same ensemble blind docking method employed to dock the first compound. Again, the docking with the smallest RMSD from the AChBP binding mode at the second agonist binding site was added to the system to create a ternary complex: nAChR saturated with two agonist molecules. Epibatidine was chosen as the agonist in the model to correspond to the agonist used in functional assays [32].

Upon creation of the ternary complex for both hα3β4 and hα4β2 nAChR models, the systems underwent one final MD simulation to create ensembles of epibatidine-bound receptor conformations. A final blind docking procedure was carried out with the antagonists illustrated in Fig. 2B. The results of the ensemble blind docking with the antagonists were clustered in the same fashion as the agonists in order to identify the most probable docking sites.

### Focused Docking and Induced Fit MD

KAB-18 was docked to focused docking grids of size 37.5 Å × 36.0 Å × 37.5 Å with 0.375 Å point spacing. The grids were centered at an α/β interface, encompassing the regions surrounding the epibatidine-bound agonist binding site. KAB-18 was docked with similar parameters as the agonists, using a cutoff of 100,000,000 energy evaluations for the LGA. Recurring docking poses were determined by clustering the docking results with an all-atom RMSD tolerance of 2 Å.

Following binding mode analysis, MD simulations of both the agonist and antagonist bound to the same subunit interface were performed to evaluate the antagonist binding stability. Two *in silico* mutant models were built with MODELLER based on a stable KAB-18 binding conformation of the hα4β2 model. Both a T58K and F118L model was built and simulated in order to correlate KAB-18 binding to these models with experimental data described below.

### Binding Energy Calculations

Binding free energies were calculated for six cases: epibatidine binding to both hα4β2 and hα3β4 nAChR models, KAB-18 binding to both models in the presence of epibatidine, and KAB-18 binding to the hα4β2 T58K and F118L models. The standard Amber MM-PBSA protocol [45] was applied to 1500 bound-state conformations, extracted at 1 ps intervals from the MD simulations described above. The receptor systems were composed of a full α/β ECD interfaces for both enthalpic and entropic calculations.

Convergence of the computed binding free energies was tracked to assure sufficient sampling. Standard deviations of time averages are reported for sliding average data with a window size of 200 data points. Time averages at increasing intervals were computed (Fig. S2) to quantify the convergence of each binding free energy. The average change in computed binding free energy between the first 1400 and first 1500 data points was 0.16 kcal/mol for the six cases reported, supporting the convergence of the values over the sampling period.

### Measurement of Intracellular Calcium Using HEK ts201 Cells Transiently Expressing Recombinant nAChRs

Calcium accumulation assays were performed as described previously [34] with slight modifications. Briefly, HEK ts201 cells, transiently expressing hα4β2WT nAChRs or hα4β2M nAChRs, were plated on 96-well plates at a density of 2.6×10^5^ cells per well. Twenty-four hours after plating, the cells were washed and then incubated with fluo-4-AM for 30 minutes at 37°C followed by 30 minutes at 24°C. After incubation, cells were washed and fluorescence was measured at ∼0.7 second intervals using a fluid handling integrated fluorescence plate reader (Flex Station, Molecular Devices, Sunnyvale, CA). The experimental design involved three treatment groups (control-sham treated, control-epibatidine treated, and antagonist treated). Functional responses were quantified by first calculating the net fluorescence changes (the difference between control sham-treated and control agonist-treated groups). Data were expressed as a percentage of control-epibatidine treated groups. Results were calculated from the number of observations (n) performed in triplicate. Curve fitting was performed by Prism software (GraphPad, San Diego, CA). EC_50_ values, IC_50_ values, and Hill coefficients were obtained by averaging values generated from each individual concentration-response curve. EC_50_ values and IC_50_ values were expressed as geometric means (95% confidence limits). Experimental values were compared using the t-test (p<0.005), as indicated.

### Site-Directed Mutagenesis and Transient Expression of nAChRs in HEK Cells

Human nAChR α4 and β2 full-length cDNAs in the vector pSP64 (poly A) were obtained from Dr. Jon Lindstrom (University of Pennsylvania) and used as the template for mutagenesis (β2) and for transient expression (α4 and β2). A single mutation was made in the β2 subunit using the Quik Change Lightning Multi Site-Directed Mutagenesis Kit (Stratagene) following the manufacturers instructions. Primers were designed using the QuikChange Primer Design Program (Stratagene) and Oligo 4.0 (National Biosciences) and synthesized by Invitrogen. Primers were designed to replace the threonine residue at position 58 in the hβ2 subunit with a lysine found at the similar position in the hβ4 subunit. The following primer was designed to change the threonine (ACC) at position 58 in the β2 subunit to lysine (AAG): β2 mutant 5′-CCACCAATGTCTGGCTGAAGCAGGAGTGGGAAGATTATCG-3′. The underlined nucleotides defined the mutation. Primers were also designed to replace the phenylalanine residue at position 118 in the hβ2 subunit with a leucine found at the similar position in the hβ4 subunit. The following primer was designed to change the phenylalanine (TTC) at position 118 in the β2 subunit to leucine (TTG): 5′-TCTCCTATGATGGTAGCATCTTGTGGCTGCCGCCTGC-3′ and 5′- GCAGGCGGCAGCCACAAGATGCTACCATCATAGGAGA-3′. It should be noted that for the F118L mutation, an additional mutation (T) was introduced which did not change the coding sequence, but relaxed a potential loop in the primer in order to allow for the generation of this mutation. The mutant hβ2 cDNAs were subcloned into pcDNA 3.1+Zeo (Invitrogen). The wild type hα4 and hβ2 cDNAs were also subcloned into the pcDNA 3.1+ and pcDNA 3.1+ Zeo vectors respectively. All cDNA clones were completely sequenced using a 3730 DNA Analyzer (Applied Biosystems) at the Ohio State University Plant-Microbe Genomics Facility. DNAs used for transfection were purified using PureLink High Pure Mini or Midi Kits (Invitrogen). HEK ts201 cells (kind gift of Dr. Rene Anand, Ohio State University Department of Pharmacology) were transiently transfected with wild-type hα4 mutant hβ2 or wild-type hα4β2 cDNAs using Lipofectamine 2000 (Invitrogen) in 60 mm dishes. After 8 hours, the cells were replated into 96 well dishes for the intracellular calcium accumulation assays.

## Results

### Homology Modeling

**Figure 3:**
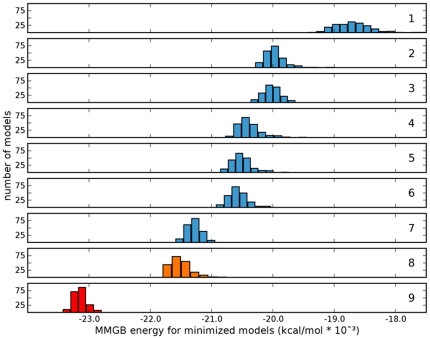
Histograms of model energies per modeling iteration. The number of models per cluster were plotted against the total calculated model energy (internal energy + PB solvation energy term) in kcal/mol * 10^−3^. Iterations 2-7 incorporates the top scoring model from the previous iteration as an additional template. **1.** Two roughly aligned AChBP templates (PDB ID: 1UWG and PDB ID: 2BYR) were used with symmetry restraints. **2.** An additional AChBP template (PDB ID: 2BJ0) was included; template alignment was refined, secondary structure assignments and distance restraints of select conserved motifs were added. **3.** β-sheet restraints were added. **4.** Mouse α1 monomer (PDB ID: 2QC1) was included as a fourth crystallographic template; α1 template specifically used to refine loop 1; hydration pocket waters added. **5.** α1 template was used to refine F loop conformation. **6.** C loop conformation of β subunits was refined. **7.** The A loop of all subunits were refined with a template modified by LES MD simulation; symmetry were restraints removed. **8.** Human α3β4 ECD models built using same alignments and constraints as in G. **9.** Human α4β2 ECD models built using same alignments and constraints as in G.

In order to identify a new allosteric site on nAChRs, it was necessary to build high-quality human nAChR models. Initially, our work began with modeling rat α3β4 nAChRs to complement experimental data related to function [33], [34]. This work served as the basis for the human nAChR modeling presented here. Figure 3 illustrates the progression of refinement through the seven iterations of rα3β4 modeling. The calculated MM-PBSA (molecular mechanics Poisson-Boltzmann/surface area) energy of the rα3β4 model was reduced by 11.2% from the initial round of modeling through the final iteration. Three modeling adjustments that made the most significant improvements in the calculated energies included refinement of the alignment with secondary structure assignments, incorporation of the mouse α1 monomer as a fourth crystallographic template, and LES (locally enhanced sampling) refinement of loop A, which yielded -4.47%, -2.45%, and -3.50% changes in total computed energy, respectively. Over the 3.93 ns duration of the simulation, the all-atom root mean square deviation (RMSD) for residues in the LES regions reached a maximum RMSD of 5.3 Å with respect to the starting structure. Comparing this deviation to the maximum RMSD of 3.4 Å exhibited by the non-LES residues characterizes the enhanced sampling achieved by this treatment.

The final hα4β2 and hα3β4 models were very similar to each other as they were both based on the same modeling restraints and share a one-to-one alignment. The backbone RMSD between the two models was 1.1 Å. Comparing the two models to the templates, the most prominent structural differences were in the conformations of loop 1 (α1-β1 loop), the A loop (β4-β5 loop), and the F loop (β8-β9 loop). The loop 1 conformations were most similar to the mouse template for the modeled α subunits, however the corresponding loops in the modeled β subunits had a unique conformation due to a different alignment. Modeled F loop conformations were most similar to the *A. californica* AChBP and mouse templates, where the modeled A loops took on conformations that placed the backbone between those of the molluskan and mammalian templates.

### Blind Docking

**Figure 4:**
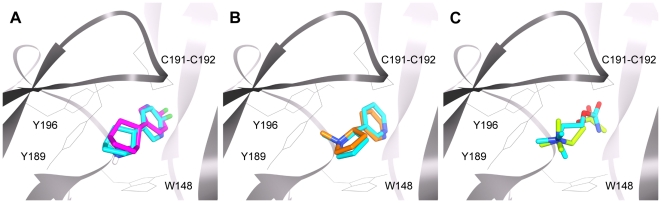
Blind docking modes compared to X-ray structures. Docking modes for epibatidine (A – magenta), nicotine (**B** – orange), and acetylcholine (**C** – green) to hα4β2 models compared to crystallographic binding modes (blue). Crystallographic structures for AChBP bound to epibatidine, nicotine, and carbamylcholine (PDB IDs: 2BYN, 1UW6, and 1UV6 respectively) were superimposed on nAChR ECD models to determine RMSDs of the dockings.

The binding site of three nAChR antagonists, COB-3, PPB-9, and APB-12 (Fig. 2B) [32], [34], was searched for using blind docking methods in conjunction with molecular dynamics simulations. Since these compounds have been shown to be non-competitive nAChR antagonists [33], [34], they were docked in the presence of agonist. Prior to antagonist docking, several agonists with known binding sites, acetylcholine, nicotine, and epibatidine (Fig. 2A), were used to test and validate the blind docking approach to the nAChR models. After docking the three agonists to 26 individual hα3β4 and hα4β2 model conformations, the results were compared to find the most frequently occurring docking positions. The docking results for the agonists near the agonist binding site are presented in Table 1 with representative docking modes illustrated in Fig. 4. Although blind docking of the agonists to the hα3β4 snapshots was able to locate both binding sites, only one of the two hα4β2 binding sites was properly located. This was due to an unusual C-loop conformation at agonist binding site 1 in the unbound state (described in Dynamics Analysis). Experimental binding affinities for all three agonists on human nAChRs could not be found in the literature, however the EC_50_ values for acetylcholine, nicotine, and epibatidine have been reported for recombinant hα4β2 and hα3β4 receptors expressed in HEK293 and *Xenopous* oocytes [46], [47], [48]. The docking energies for the agonists were able to reproduce binding energy trends, with epibatidine binding more strongly than nicotine which displays greater binding affinity than acetylcholine. Additionally, the average docking energies of the agonists all showed a preference to bind the hα4β2 models over the hα3β4 models, a trend that is also experimentally observed [46], [47], [48].

**Table 1:** Blind docking results for agonists to multiple hα4β2 and hα3β4 nAChR ECD conformations.

	hα4β2				hα3β4			
	average docking energy (kcal/mol)^a^	expt. potency, EC_50_ (µM)^b^	cluster size	average RMSD of dockings (Å)^c^	average docking energy(kcal/mol)^a^	expt. potency, EC_50_ (µM)^b^	cluster size	average RMSD of dockings (Å)^c^
acetylcholine	−4.86	100	132	3.13	−4.66	203.14	150	6.45
nicotine	−6.59	3.5	282	1.72	−6.31	40.3	90	4.97
epibatidine	−7.83	0.043	154	5.44	−7.28	0.151	149	7.42

a AutoDock energies.

b Experimental agonist potencies from data reported in [46], [47], [48].

c RMSD measurements with respect to corresponding AChBP crystal complexes

Three antagonists, COB-3, PPB-9, and APB-12, were docked to the models using the same ensemble blind docking method that was used to dock the agonists. Based on LigPrep (Schrödinger, LLC) results, the antagonists are all positively charged at physiologic pH, protonated at the nitrogen atom of their piperidine/pyrrolidine moieties. Each antagonist also has one or more stereogenic centers. The two stereoisomers of each compound with the lowest computed energy were used in the blind docking study; each of these conformations had equatorial branching off of the heterocyclic moieties. The antagonist docking site that was ultimately validated as the correct binding site was populated by 28.2% of the dockings to the epibatidine-bound model conformations. Three other sites were more prominently populated with alternate docking clusters; these had 69.6%, 57.1% and 47.1% rates of being identified as one of the four largest docking clusters for each antagonist that was docked. The positions of these other sites were all located on the inside of the doughnut-shaped extracellular domain facing the pore. They were either at subunit interfaces (both α/β and β/β) or tucked inside an A loop.

**Figure 5:**
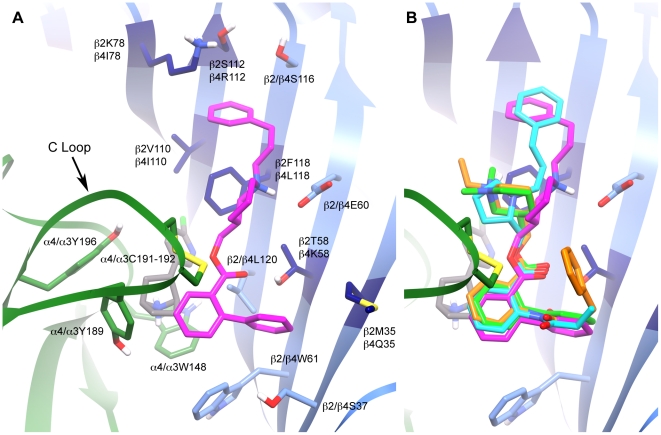
Detailed antagonist docking modes. **A.** Docking mode of KAB-18 (magenta) at the α4(+) (green)/β2(-) (blue) interface in the presence of the agonist epibatidine (grey). Residues varying between the β2 and β4 subunits are featured (dark blue). **B.** Superimposed Glide docking modes of KAB-18 (magenta), APB-12 (cyan), PPB-9 (orange), and COB-3 (green) at the same binding site.

One of the frequently occurring antagonist blind docking modes was investigated more closely by redocking the antagonist KAB-18 to the new allosteric site with focused docking grids. KAB-18 became a focus because this compound exhibits preferential antagonism of hα4β2 nAChRs versus hα3β4 nAChRs [32]. The selection of a precise docking mode was aided by existing structure activity relationship (SAR) data that indicate modifying the terminal phenyl of the biphenyl group of KAB-18 to a succinimide moiety results in a loss of hα4β2 selectivity [46]. Additionally, modifying the length of the aliphatic linker on the opposite end of the antagonist was also shown to result in a loss of relative hα4β2 selectivity. Taking this into consideration, a binding mode in which the aforementioned regions of KAB-18 were found to associate with receptor residues that vary between the hα4β2 and hα3β4 nAChRs was selected. This mode is illustrated in Fig. 5A, highlighting the amino acids with which the antagonist makes contact, while a superposition of the other antagonist docking modes is found in Fig. 5B. Interestingly, the residues that seem to confer selectivity for this binding mode, i.e. those sites of variation between the hα4β2 and hα3β4 subtypes (amino acids at positions 78, 110, 112, 118, 58, and 35), are all found on the β subunit, forming a band along the 6-membered β-sheet that creates the (-) side of the α/β interface (dark blue in Fig. 5A).

### Dynamics Analysis and Hypothetical Mode of Antagonism

Since that antagonists we are studying act allosterically, it was important to model the receptor in the presence of agonist as would occur *in vivo.* To prepare the nAChR models for antagonist blind docking, MD simulations were conducted for the receptors in various binding states, including an unbound state, a binary complex bound to a single epibatidine molecule, and a ternary complex saturated with two epibatidine molecules. The stability of these simulations was quantified by all-atom RMSD analysis. It was found that the Cys loops were conformationally unstable, leading to steadily increasing RMSDs over the duration of the 5 ns simulations. However, when the RMSDs were recalculated to exclude the Cys loop residues, the all-atom RMSD for each model leveled off in the range of 2–3 Å, indicating stable MD trajectories (Fig. S3).

The average RMSDs from the starting structures for each subunit in the three sampled binding states showed that the MD trajectories were relatively stable (Fig. S4, Table S3). The maximal backbone RMSD average for a single subunit was 4.56 Å, while the typical subunit only deviated an average of 2.02 Å from its initial conformation over simulation times of 5 ns. Some regions, including the C, Cys, and L1 loops, were particularly more variable in conformation when compared to each subunit as a whole, while the A, B, and F loops were generally more stable. Plots of the all-atom RMSDs on a per residue basis are shown in Fig. S4.

Most nAChR agonists, including epibatidine, carry a positive charge at physiologic pH. This plays a significant role in their binding to the nAChRs due to cation-π interactions between a group of aromatic residues at the agonist binding site and the positively charged agonist [49]. In addition to cation-π interactions, proper fitting into the agonist binding site can allow for strong hydrogen bond formation between the positively charged nitrogen of the agonist and the backbone carbonyl of Trp148, as observed in crystallographic structures [35], [36] and proven important in mutational studies [49]. Both of these interactions have been measured in our dynamics studies (Table 2, Figs. S5 and S6 for plots of distance measurements). Based on these measurements, the epibatidine molecules were found to be stably bound in the agonist binding sites of the models over the 5 ns MD simulations. However, introduction of a second epibatidine molecule to the hα4β2 model resulted in a 90° torsion around the one freely rotating bond of the epibatidine molecule, causing a temporary break in the hydrogen bond with Trp148 (Figure S6B). This hydrogen bond is ultimately reformed for the final 2 ns of the MD simulation.

**Table 2:** Measurements of agonist binding distances in MD simulations of epibatidine bound hα4β2 and hα3β4 nAChR ECDs.

	hα4β2		hα3β4	
	agonist binding site 1	agonist binding site 2	agonist binding site 1	agonist binding site 2
hydrogen bonding interaction distance (Å)^a^				
binary complex^c^	2.88 (0.13)	–	2.84 (0.11)	–
ternary complex^d^	2.89 (0.13)	3.75 (1.16)	2.84 (0.12)	2.86 (0.13)
cation-π interaction distance (Å)^b^				
binary complex^c^	3.91 (0.39)	–	3.34 (0.25)	–
ternary complex^d^	3.72 (0.38)	4.55 (0.75)	3.45 (0.27)	4.87 (0.49)

a Distance between positively charged N of epibatidine and backbone O of Trp148.

b Distance between positively charged N of epibatidine and center of mass for the indole group of Trp148 (Å).

c Single epibatidine molecule bound to agonist binding site 1.

d Epibatidine bound to both agonist binding sites.

Average measurements calculated from 5 ns MD simulations with standard deviations in parentheses.

**Figure 6:**
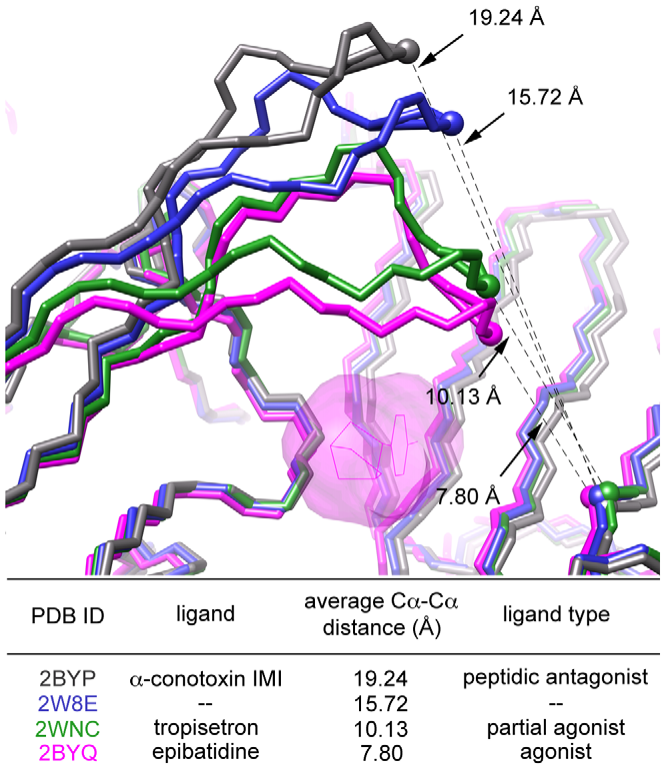
C loop closure of AChBP bound to various ligands. Superposition of four crystal structures of AChBP in complex with various compounds to illustrate the difference in intersubunit distances between Cα of residue C191 of the α subunit C loop on the (+) side of the binding interface and Cα of residue 58 of the β subunit β2 strand on the (−) side of the interface. Only epibatidine is shown (pink surface) for clarity, to highlight the ligand binding site. The tabulated Cα-Cα distances allows for quantification of the degree of C loop closure upon ligand binding.

C loop dynamics are another important aspect of ligand binding to the agonist binding site of nAChRs as these motions are thought to initiate channel gating [22]. As observed in crystallographic structures, the C loop takes on a ‘closed’ or capped conformation upon binding of small agonists such as nicotine, acetylcholine, or epibatidine, while competitive antagonists are much larger than agonist compounds, and their presence in the binding site can force the C loop into a more ‘open’ conformation [36]. To track C loop dynamics in our MD simulations, we measure the Cα-Cα distance between αCys191 on the (+) side of the binding site and β58 (β2Thr58/β4Lys58) on the (−) side of the interface as illustrated in Fig. 6. These corresponding distances for 22 different AChBP crystal structures were measured (Table S4) and have been used to create generalized Cα-Cα ranges of C loop ‘openness’ for agonist, partial agonist, and antagonist binding in addition to unbound states which are grouped with the non-peptidic antagonists (Table 3). The general range for agonist binding, based on four X-ray structures, is 7.72–8.19 Å, compared to the unbound state which has a range of 15.36–15.72 Å based on two structures.

**Table 3:** General ranges for C loop “openness” upon binding ligands of different pharmacological function measured from AChBP X-ray structures.

	average Cα-Cα range (Å)^a^
agonist	7.72–8.19
partial agonist	9.75–12.30
antagonist/unbound	12.88–16.05
peptidic antagonist	17.50–19.24

a Average Cα-Cα distance between residues that correspond to C191 on the C loop of nAChR α subunits on the (+) side of the binding interface and residue 58 on the β2 strand of β subunits on the (−) side of the interface.

Using this Cα-Cα distance between the tip of the C loop and the β2 strand of the adjoining subunit as a metric of C loop closure, we have been able to quantify these dynamics (Table 4) and relate them to different functional states (Table 3). The average Cα-Cα distance from the 5 ns MD simulations of unbound agonist binding sites (apo binding sites 1 & 2, binary complex binding site 1) all had values between the partial agonist and unbound ranges defined in Table 3, implying more “open” C loops (see Figs. S7 and S8 for plots of Cα-Cα distances collected from MD trajectories). An exception was observed for agonist binding to site 1 of the apo hα4β2 receptor. Here, the C loop is closed in the absence of agonist, which may represent the closed unbound state observed by Mukhtasimova et al [50]. Upon agonist binding, the measured Cα-Cα distances decreased to values in the ranges measured for agonist-bound and partial agonist-bound receptors (Table 3), consistent with structural data that implicates agonists causing C loop closure to initiate channel opening [36]. In the bound states, the low standard deviations indicate relatively stable C loop conformations; the standard deviations for the time-averaged Cα-Cα distances are greater in the unbound states.

**Table 4:** Measurements of C loop closure for MD simulations of epibatidine bound hα4β2 and hα3β4 nAChR ECDs.

	average Cα-Cα distance (Å)^a^			
	hα4β2		hα3β4	
	agonist binding site 1	agonist binding site 2	agonist binding site 1	agonist binding site 2
apo	8.94 (1.57)	15.08 (1.73)	12.68 (2.12)	15.06 (2.25)
binary complex^b^	7.78 (0.46)	14.32 (3.00)	8.69 (0.48)	12.99 (1.60)
ternary complex^c^	8.02 (0.45)	11.62 (1.79)	10.62 (1.35)	7.88 (0.38)
	average distance (Å)	minimum distance (Å)	average distance (Å)	minimum distance (Å)
epibatidine/KAB-18 complex^d^	12.97 (1.49)	10.58	18.97 (2.95)	11.55

a Same Cα-Cα measurement as defined in Table 4.

b single epibatidine molecule bound to agonist binding site 1.

c epibatidine bound to both agonist binding sites.

d compounds bound to agonist binding site 2.

Data averaged over 5 ns MD simulations with standard deviations in parentheses.

In our computational KAB-18 binding studies, the dynamics of the C loop show that even though epibatidine is forming a stable hydrogen bond with the carbonyl oxygen of Trp148, the C loop is obstructed from closing to an agonist-bound state due to the presence of KAB-18. The minimum Cα-Cα distances in simulations of epibatidine and KAB-18-bound hα4β2 and hα3β4 nAChRs was 10.58 and 11.55 Å respectively, while the average values over 5 ns of simulation were larger at 12.97 and 18.97 Å respectively.

### Binding Energy Calculations

The binding free energies for several ligand binding events to the nAChR ECD models have been calculated with the MM-PBSA protocol in Amber [45]. The free energies for epibatidine binding alone and KAB-18 binding in the presence of epibatidine were calculated for both hα4β2 and hα3β4 nAChR models over simulation times of 1.5 ns, with data calculated at 1 ps intervals (Table 5 and Figs. S9 and S10). A binding energy of -17.46 kcal/mol for epibatidine binding alone to the hα4β2 model was computed, compared to the experimental range of -14.49 – -14.27 kcal/mol [47]. For epibatidine binding to the hα3β4 model, a binding energy of -14.91 kcal/mol was computed, compared to the experimental range of -13.19 – -13.19 kcal/mol [48]. These more computationally intensive free energy calculations yield numbers that follow the experimental binding trends for epibatidine in addition to being much closer estimates of the experimentally derived energies than the AutoDock scores reported in Table 1. The binding energy calculations for KAB-18 binding to the hα4β2 nAChR model showed a favorable binding energy of -6.25 kcal/mol, while an unfavorable binding energy of 11.25 kcal/mol was calculated for KAB-18 binding to the hα3β4 nAChR model.

**Table 5:** MM-PBSA binding energy calculations for epibatidine and KAB-18 bound to hα4β2 and hα3β4 nAChR ECD models.

	hα4β2-WT	hα4β2-T58K	hα4β2-F118L	hα3β4-WT
epibatidine binding^a^				
ΔH	-33.28 (1.01)	–	–	-32.29 (0.96)
TΔS	-15.82 (2.07)	–	–	-17.37 (1.28)
ΔG	-17.46 (2.32)	–	–	-14.91 (1.46)
Expt. range^b^	-14.49 – -14.27	–	–	-13.25 – -13.19
distance 1^c^	2.86 (0.04)	–	–	2.83 (0.02)
distance 2^d^	7.79 (0.30)	–	–	8.65 (0.27)
KAB-18 binding in presence of epibatidine^e^				
ΔH	-28.27 (2.02)	-28.56 (2.69)	-21.30 (1.67)	-14.90 (0.98)
TΔS	-22.02 (1.77)	-23.22 (1.99)	-28.61 (2.28)	-26.15 (2.38)
ΔG	-6.25 (2.86)	-5.34 (3.32)	7.31 (2.59)	11.25 (3.06)
distance 1^c^	2.90 (0.06)	2.93 (0.07)	2.97 (0.11)	2.86 (0.03)
distance 2^d^	11.83 (0.21)	13.76 (0.40)	16.84 (0.34)	13.61 (0.57)

a Binding at agonist binding site 2 in the absence of antagonist.

b Experimental binding affinities calculated from data reported in [47], [48].

c Distance between the positively charged N atom in the bound epibatidine molecule and the backbone O atom of Trp148, quantifying epibatidine binding stability.

d Cα-Cα distance between α191 and β58 at the binding interface, quantifying C loop closure.

e Both compounds bound at agonist binding site 2.

Data averaged over 1.5 ns MD simulations with standard deviations in parentheses.

The binding energy for KAB-18 bound to two hα4β2 models with mutations in the putative allosteric binding site were also assessed with the MM-PBSA method. KAB-18 was computed to bind slightly weaker to the model with a T58K mutation on the β2 subunit with binding energy of -5.34 kcal/mol, a 0.91 kcal/mol difference from the wild-type binding energy. A F118L mutation on the β2 subunit resulted in a positive computed binding energy of 7.31 kcal/mol. Both of these *in silico* mutation experiments correspond with the functional data presented below.

### Mutagenesis

To experimentally validate the computationally determined KAB-18 binding mode, two independent mutations were made on the β2 subunit of α4β2 nAChRs expressed in HEK cells. Functional IC_50_ values fro KAB-18 and control antagonists (d-tubocurarine and mecamylamine) as well as function EC_50_ values for a control agonist (epibatidine) were obtained using a fluorescence calcium accumulation assay. Changes in the IC_50_ values of KAB-18 were used to document a change in the apparent affinity of KAB-18 as caused by mutation of the target amino acids. First, threonine at position 58 was selected for mutation based on its contribution of a key hydrogen bonding donor to the binding stability of the theoretical binding mode presented in Fig. 5A. Lysine was chosen to replace threonine since this amino acid is found at the same position on human β4 subunits and KAB-18 has no functional activity on human α3β4 nAChRs when tested at concentrations up to 100 µM [32] (higher concentrations were not possible due to solubility limitations). The IC_50_ for KAB-18 was reduced to 71.8 µM on the β2T58K mutant from the wild-type IC_50_ of 8.5 µM (Table 6 and Fig. S11), an eight-fold decrease in observed potency. Second, the phenylalanine at position 118 was mutated to leucine. As in the case of the T58K mutation, the phenylalanine was mutated to leucine as it is the corresponding residue on the human β4 subunit. This mutation resulted in a loss of inhibitory activity for KAB-18 at concentrations up to 100 µM (Table 6 and Fig. S11). It is important to note these single point mutations did not affect apparent affinities at either 1) the orthosteric site for epibatidine (an agonist) and tubocurarine (a competitive antagonist) or 2) the binding site for mecamylamine, a non-competitive antagonist which binds at a different location on the receptor.

**Table 6:** Effects of agonists and antagonists on wild-type and mutated human α4β2 nAChRs.

	hα4β2-WT nAChRs		hα4β2-T58K nAChRs		hα4β2-F118L nAChRs	
	EC_50_ or IC_50_ Values^a^	*n*_H_ b	EC_50_ or IC_50_ Values^a^	*n*_H_ b	EC_50_ or IC_50_ Values^a^	*n*_H_ b
epibatidine (EC_50_)^c^	33.9 (20.1–57.2) nM	0.9	29.2 (9.8–87.2) nM	0.7	23.7 (12.6–44.6) nM	0.8
*d*-tubocurarine (IC_50_)	6.3 (4.0–10.0) µM	−1.0	6.2 (2.1–18.5) µM	−0.6	6.5 (3.9–10.9) µM	−0.9
mecamylamine (IC_50_)	0.2 (0.1–0.4) µM	−1.4	0.2 (0.1–0.5) µM	−0.6	0.4 (0.3–0.5) µM	−1.1
KAB-18 (IC_50_)	8.5 (5.4–13.4) µM	−1.2	71.8 (48.3–107.3) µM^c^	−1.0	>100 µM^d^	−-

a Values represent geometric means (confidence limits), n = 5-7.

b *n*_H_, Hill coefficient.

c significantly different from wild-type response, p<0.005.

d compound is insoluble at concentrations greater than 100 µM.

Data ranges in parenthesis.

## Discussion

Nicotinic acetylcholine receptors serve as a prototype for ligand-gated ion channels and are one of the most studied allosteric membrane proteins [51]. In this study, we identified the binding site of a negative allosteric modulator of hα4β2 nAChRs called KAB-18. *In silico* modeling, docking, MD simulations, and binding energy calculations were used to predict the binding mode while site-directed mutagenesis and functional assays provided experimental data that supported the theoretical model.

The iterative homology modeling approach was shown to be successful in refining the nAChR models, with each successive iteration reducing the computed receptor energies (Fig. 3). Conventionally, nAChR models are based on a single template [23], [24], [25], [26], [27], [28], [29], while the models reported in this paper are based on four crystallographic templates. Incorporating the mouse α1 monomer into the homology modeling process refined the conformation of several loop regions: L1, L5 (A loop), L7 (Cys-loop), and L9 (F loop) due to the one-to-one sequence alignment found in the mouse that is not present in molluskan AChBP. In addition to template differences, the alignment used to create the models in this paper is unique, particularly in loop regions, from those previously reported. This implies differences in model structure that likely effect docking and dynamics results.

Flexibility of the agonist binding site has been documented by unbound and agonist-bound AChBP crystal structures [36]. When docking, this flexibility was accounted for by the use of multiple receptor conformations as extracted from MD trajectories. The AChBP structures suggest that ligands induce a conformational change of the C loop upon binding. Thus, the high RMSD values for agonist docking (Table 1), which are in reference to the binding modes found in AChBP crystal structures, may be explained by the expected induced-fit effect between the ligand and receptor. Docking to the multiple receptor conformations helped locate the correct binding site. However with only docking data, it would have been difficult to distinguish the proper docking site from the false positives. SAR data have shown some compounds in this class of antagonists to selectively act on α4β2 over α3β4 nAChRs [32] implying that the correct docking mode would interact with residues that were not conserved between the α4β2 and α3β4 nAChRs. Ultimately, two of these non-conserved residues were mutated to experimentally validate the binding site in functional assays.

Quantification of the C loop dynamics for the KAB-18-bound models (Table 4) coupled with the large set of structures examining AChBP bound to numerous ligands of varied pharmacological effect (Table 3, Fig. 6, and Table S4), indicates a possible mechanism of noncompetitive antagonism: inhibition of C loop closure that is required for the channel to open while not interfering with agonist binding. Although this mode of antagonism has been previously noted [36], [52], this is the first time a negative allosteric modulator has been suggested to act in this fashion.

**Figure 7:**
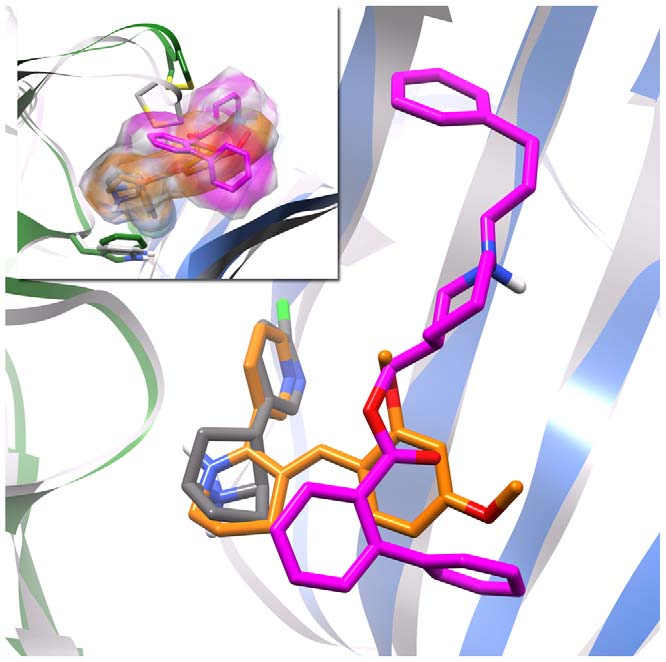
Comparison of experimental DMXBA binding to computationally predicted KAB-18/epibatidine binding. The X-ray structure of DMXBA (orange) in complex with *Aplysia californica* AChBP (grey ribbon) superimposed on a hα4β2 nAChR ECD model (green and blue ribbon for α4 and β2 subunits, respectively) bound to both epibatidine (grey) and the negative allosteric modulator KAB-18 (magenta). The C loops for each protein have been removed for clarity in the main figure, while the inset features the varied degree of C loop closure.

Furthermore, superposition of the X-ray structure of AChBP in complex with the α7 nAChR partial agonist, 3-(2,4-dimethoxybenzylidine)-anabaseine (DMXBA) [53], to a MD snapshot of our equilibrated epibatidine and KAB-18-bound hα4β2 nAChR complex, reveals interesting similarities in ligand binding (Fig. 7). The anabaseine portion of DMXBA superimposes well with the epibatidine molecule bound in the nAChR model, while the dimethoxybenzylidine moiety of DMXBA branches towards the (-) surface of the subunit interface to the same region occupied by KAB-18 in the nAChR model. Anabaseine acts as a full α7 agonist, while the addition of the dimethoxybenzylidine group reduces the level of efficacy, transforming the molecule into a partial agonist [53]. The experimental Cα-Cα measurements of C loop closure for anabaseine average 7.72 Å in the bound state while DMXBA measures 9.75 Å. KAB-18 seems to share some of the nAChR binding qualities that make DMXBA antagonists, However KAB-18 is able to more effectively prevent C loop closure while not competing with the agonist binding site. These similar binding features coupled with varied degrees of C loop closure can provide some insight on what may differentiate a partial agonist from a full agonist or antagonist; pharmacological effects of a ligand binding at or near the orthosteric site are related to the degree to which the ligand induces or inhibits C loop closure.

Finally, to experimentally validate our binding mode prediction, the pharmacological activity of KAB-18 was tested on hα4β2WT, hα4β2M T58K, and hα4β2M F118L nAChRs. Our prediction, that disrupting the hydrogen bond formed between KAB-18 and side chain of T58 would decrease the apparent affinity of the compound for the receptor, was validated when an eight-fold decrease in potency was measured in a functional assay (Table 6). The change in potency of KAB-18 is indicative of an observed change in its apparent affinity for the receptor due to the T58K mutation, supporting the involvement of T58 in the binding of KAB-18. This experimental result was backed up by an *in silico* mutation which showed that KAB-18 still bound stably to the mutant receptor, and even formed a hydrogen bond with the lysine side chain. However, due to the mobility of lysine residues, this hydrogen bond was weaker and shorter lived than the hydrogen bond formed with the threonine side chain in the wild-type receptor.

The F118L mutation resulted in a loss of inhibitory activity for KAB-18 up to concentrations of 100 µM (Table 6). As with the T58K mutation, this result is indicative of a change in apparent affinity of KAB-18 and supports the involvement of F118 in the binding of KAB-18. The observation that agonist activity was unaffected by this mutation (Table 6) shows that L118 altered KAB-18 binding while leaving the receptor functionally intact. The simulation of KAB-18 in the allosteric binding site of the F118L model revealed that the weakened binding could be due to the loss of a π-π stacking interaction between the F118 side chain and the terminal phenyl group of KAB-18 in addition to the loss of a cation-π interaction between the F118 side chain and the positively charge piperidine moiety of KAB-18. These data support the experimental findings that KAB-18 preferentially inhibits hα4β2 over hα3β4 nAChRs [32] and are consistent with KAB-18 binding to the allosteric site predicted by computational modeling. This allosteric site can be used to develop drugs targeted to specific nAChR subtypes.

## Supporting Information

Figure S1 **Numbered sequence alignment of AChBP and nAChR sequences used for modeling.** Templates (bold) are the acetylcholine binding protein from three molluskan species (*Lymnaea stagnalis*, *Aplysia californica*, and *Bulinus truncatus*) and the mouse α1 nAChR ECD. Targets are the human α3, α4, β2, and β4 nAChR ECDs. Magenta highlighting indicates a conserved residue, while turquoise highlighting indicates residue similarity. Light green bars above residues represents α helices, dark green bar represent 3_10_ helices, and light blue arrows represent β strands. The alignment was done manually with cues taken from AChBP X-ray structures and the secondary structure prediction algorithms PHD and PSIPRED.
(TIF)

Figure S2 **Convergence of MM-PBSA calculations.** Average free energies of binding as a function of sampling period for **A.** epibatidine binding hα4β2 model **B.** epibatidine binding to hα3β4 model **C.** KAB-18 binding to epibatidine-bound hα4β2 model **D.** KAB-18 binding to epibatide-bound hα4β2 T58Kβ2 model **E.** KAB-18 binding to epibatidine-bound hα4β2 F188L model **F.** KAB-18 binding to epibatidine-bound hα3β4 model. Energies are presented as averages with ps intervals.
(TIF)

Figure S3 **RMSD plots for nAChR model MD simulations.** All-atom RMSD plots for hα4β2 (**A**) and hα3β4 (**B**) in three different states: unbound, binary complex, and ternary complex. Dashed lines represent RMSD values for the entire extracellular domain models, while the solid lines represent the RMSD for the entire models excluding the Cys loop residues. Data was smoothed with a 50 frame sliding window average.
(TIF)

Figure S4 **Average all-atom RMSDs for hα4β2 and hα3β4 nAChR ECD models in three different binding states.** All-atom RMSD of each residue from the initial structure of a 5 ns MD simulation of three states: unbound (blue), bound to one epibatidine molecule at agonist binding site 1 (αx_1_/βx_1_ interface) (green), and bound to an epibatidine molecule at both agonist binding sites (red). Several loop regions are highlighted, including L1 (14-27), Cys-loop (127-138), F loop (159-174), and the α-subunit C loop (189-195). **A**. hα4β2 nAChR data **B**. hα3β4 nAChR data.
(TIF)

Figure S5 **Measurement of epibatidine binding distances to hα4β2 and hα3β4 nAChR ECD binary complexes.** Distance measurements quantifying epibatidine binding stability to agonist binding site 1 for hα4β2 (**A**, **C**) and hα3β4 (**B**, **D**) nAChR ECDs from 5 ns MD simulations. The distances between the positively charged nitrogen atom of epibatidine and both the backbone carbonyl oxygen atom of Trp148 (**A**, **B**) and the center of mass for the indole group of Trp148 (**C**, **D**) are measured. Picosecond interval data are plotted in the lighter color, while sliding average data with a window size of 50 data points are plotted in the darker color.
(TIF)

Figure S6 **Measurement of epibatidine binding distances to hα4β2 and hα3β4 nAChR ECD ternary complexes.** Distance measurements quantifying epibatidine binding stability to agonist binding site 1 (**A**, **C**, **E**, **G**) and binding site 2 (**B**, **D**, **F**, **H**) for hα4β2 (**A**, **B**, **C**, **D**) and hα3β4 (**E**, **F**, **G**, **H**) nAChR ECDs from 5 ns MD simulations. The distances between the positively charged nitrogen atom of epibatidine and both the backbone carbonyl oxygen atom of Trp148 (**A**, **B**, **E**, **F**) and the center of mass for the indole group of Trp148 (**C**, **D**, **G**, **H**) are measured. Picosecond interval data are plotted in the lighter color, while sliding average data with a window size of 50 data points are plotted in the darker color.
(TIF)

Figure S7 **Measurements of C loop closure for hα4β2 and hα3β4 nAChR ECDs bound to epibatidine.** Distance data that quantifies C loop dynamics upon agonist binding to the human α4β2 nAChR extracellular domains (**A**, **B**, **C**) and human α3β4 nAChR extracellular domains (**D**, **E**, **F**). The distance between Cα atoms of C191 on the C loop of α subunits on the (+) side of the binding interface and residue 58 on the β2 strand of β subunits on the (-) side of the interface is measure for unbound states (**A**, **D**), binary complexes (**B**, **E**), and ternary complexes (**C**, **F**). Distances are given at ps intervals (light-colored plots) and are also represented as sliding averages (dark-colored plots) with a window size of 50 data points. The magenta dashed lines are the average values for each plot.
(TIF)

Figure S8 **Measurements of C loop closure for hα4β2 and hα3β4 nAChR ECDs bound to both epibatidine and KAB-18.** The distance between Cα atoms of C191 on the C loop of α subunits on the (+) side of the binding interface and residue 58 on the β2 strand of β subunits on the (-) side of the interface is measure for the hα4β2 (A) and hα3β4 nAChR ECDs bound to both epibatinde and KAB-18 at binding site 2. Distances are given at ps intervals (light-colored plots) and are also represented as sliding averages (dark-colored plots) with a window size of 50 data points. The magenta dashed lines are the average values for each plot.
(TIF)

Figure S9 **MMPB-SA binding energy calculations for epibatidine and KAB-18 binding.** Binding energy components for epibatidine binding alone (**A**, **C**) and KAB-18 binding in the presence of epibatidine (**B**, **D**) to both hα4β2 (**A**, **B**) and hα3β4 (**C**, **D**) nAChR ECDs. Data is plotted at ps intervals (light trace) in addition to a sliding average trace (dark) with a window size of 200 data points.
(TIF)

Figure S10 **MMPB-SA binding energy calculations and dynamics analysis for ligand binding to hα4β2 and hα3β4 nAChRs.** **A**. Epibatidine binding **B**. KAB-18 binding in the presence of epibatidine. The top three plots in each figure contain binding free energy data computed with the MM-PBSA protocol in Amber: ΔH (blue), TΔS (red), ΔG (purple). The bottom plot in each figure is distance data extracted from the MD simulations over the sampling period: distance between positively charged nitrogen atom of epibatidine and the backbone oxygen atom of Trp148 (green), and the Cα-Cα between α191 and β58 (yellow). The solid trace represents data for ligands bound to the hα4β2 nAChR extracellular domain model, while the dashed trace represents the hα3β4 nAChR data. All data is presented as sliding-window averages with a window size of 200 data points.
(TIF)

Figure S11 **Dose-response curves for epibatidine and KAB-18 on wild-type and mutant hα4β2 nAChRs.** **A.** Functional response for epibatidine binding to hα4β2WT (wild-type) and hα4β2M T58K/F118L mutant nAChRs. **B.** Functional response of KAB-18 on wild-type and mutant nAChRs. Data are expressed as a percentage of control responses using 3 µM epibatidine. Values represent means ± SEMs (n = 5 – 7).
(TIF)

Table S1 **Sequence identity between template and target sequences.**
(DOC)

Table S2 **Sequence similarity between template and target sequences.**
(DOC)

Table S3 **Average RMSDs for backbone atoms of α4β2 and α3β4 nAChR ECD models from MD simulations in three states.**
(DOC)

Table S4 **Survey of C loop closure for AChBP X-ray structures.**
(DOC)
