# Supplementary figures and images for: Identification of a Negative Allosteric Site on Human α4β2 and α3β4 Neuronal Nicotinic Acetylcholine Receptors
Source: PLoS One. 2011 Sep 15;6(9):e24949. doi: 10.1371/journal.pone.0024949 (PMC3174232; doi:10.1371/journal.pone.0024949)

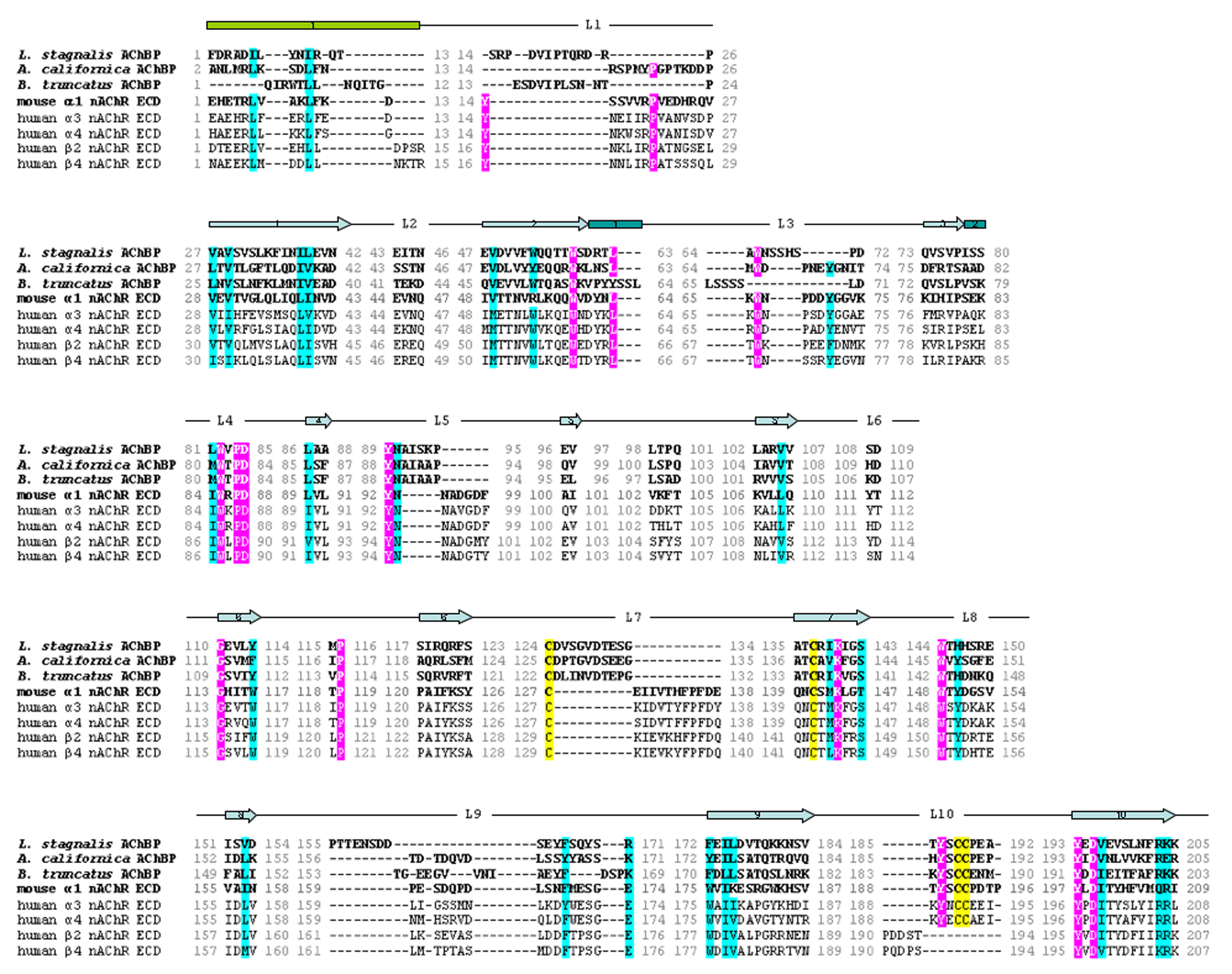

Supplement: Figure S1 — Numbered sequence alignment of AChBP and nAChR sequences used for modeling. Templates (bold) are the acetylcholine binding protein from three molluskan species (Lymnaea stagnalis, Aplysia californica, and Bulinus truncatus) and the mouse α1 nAChR ECD. Targets are the human α3, α4, β2, and β4 nAChR ECDs. Magenta highlighting indicates a conserved residue, while turquoise highlighting indicates residue similarity. Light green bars above residues represents α helices, dark green bar represent 310 helices, and light blue arrows represent β strands. The alignment was done manually with cues taken from AChBP X-ray structures and the secondary structure prediction algorithms PHD and PSIPRED. (TIF) [file pone.0024949.s001.tif]

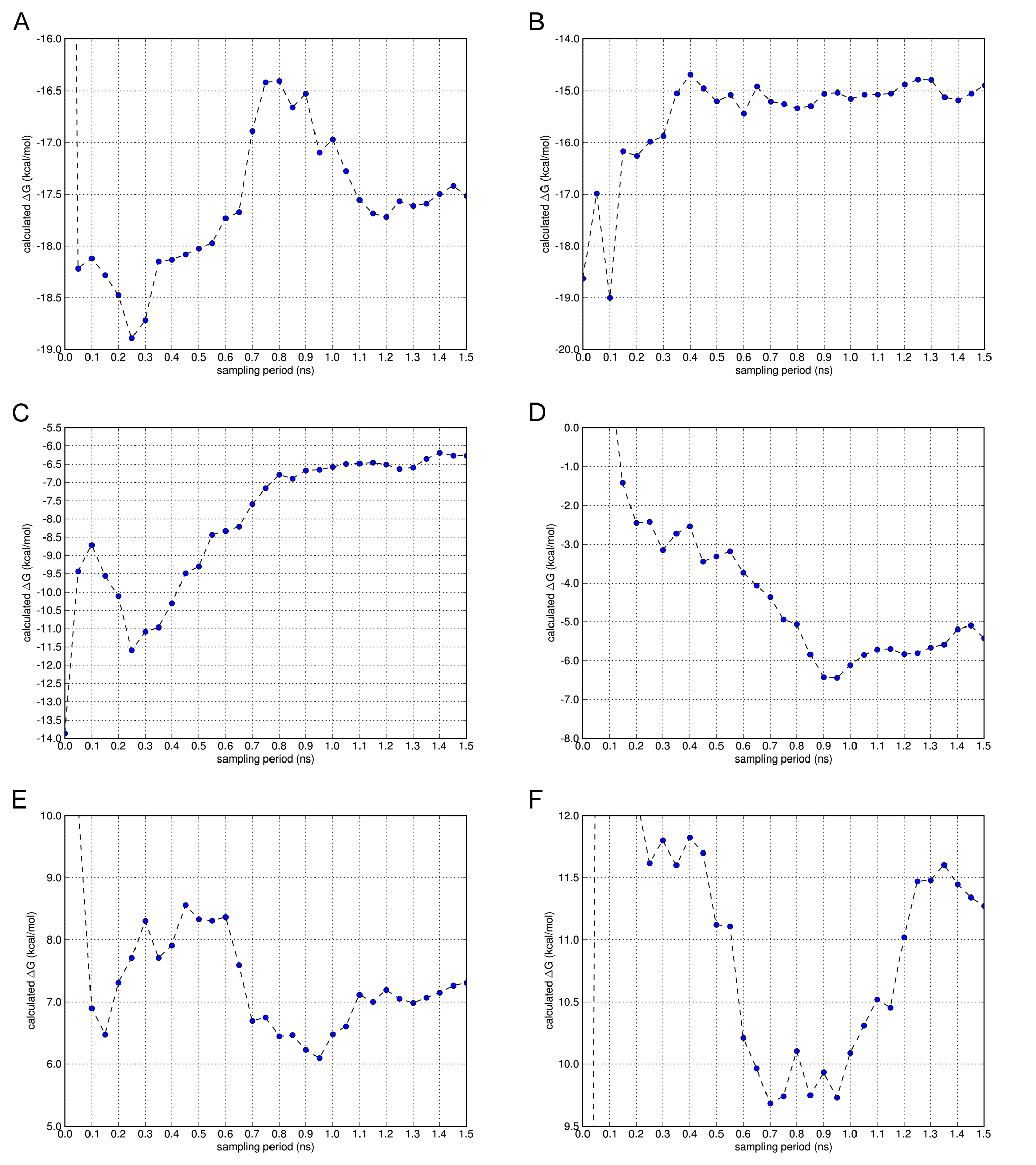

Supplement: Figure S2 — Convergence of MM-PBSA calculations. Average free energies of binding as a function of sampling period for A. epibatidine binding hα4β2 model B. epibatidine binding to hα3β4 model C. KAB-18 binding to epibatidine-bound hα4β2 model D. KAB-18 binding to epibatide-bound hα4β2 T58Kβ2 model E. KAB-18 binding to epibatidine-bound hα4β2 F188L model F. KAB-18 binding to epibatidine-bound hα3β4 model. Energies are presented as averages with ps intervals. (TIF) [file pone.0024949.s002.tif]

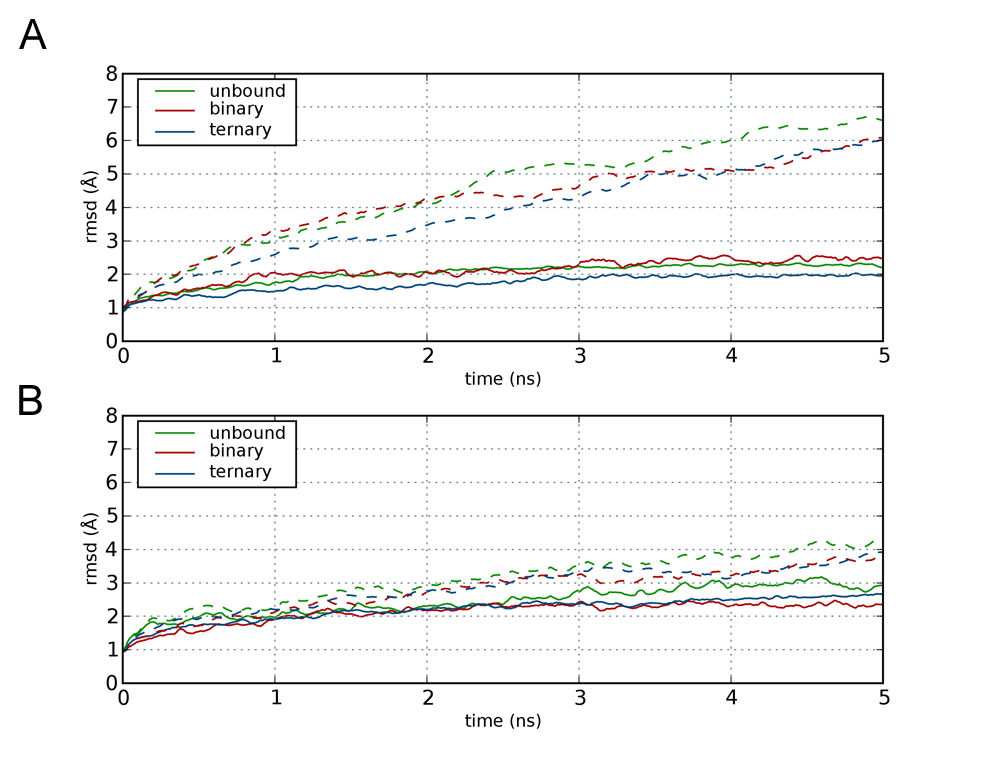

Supplement: Figure S3 — RMSD plots for nAChR model MD simulations. All-atom RMSD plots for hα4β2 (A) and hα3β4 (B) in three different states: unbound, binary complex, and ternary complex. Dashed lines represent RMSD values for the entire extracellular domain models, while the solid lines represent the RMSD for the entire models excluding the Cys loop residues. Data was smoothed with a 50 frame sliding window average. (TIF) [file pone.0024949.s003.tif]

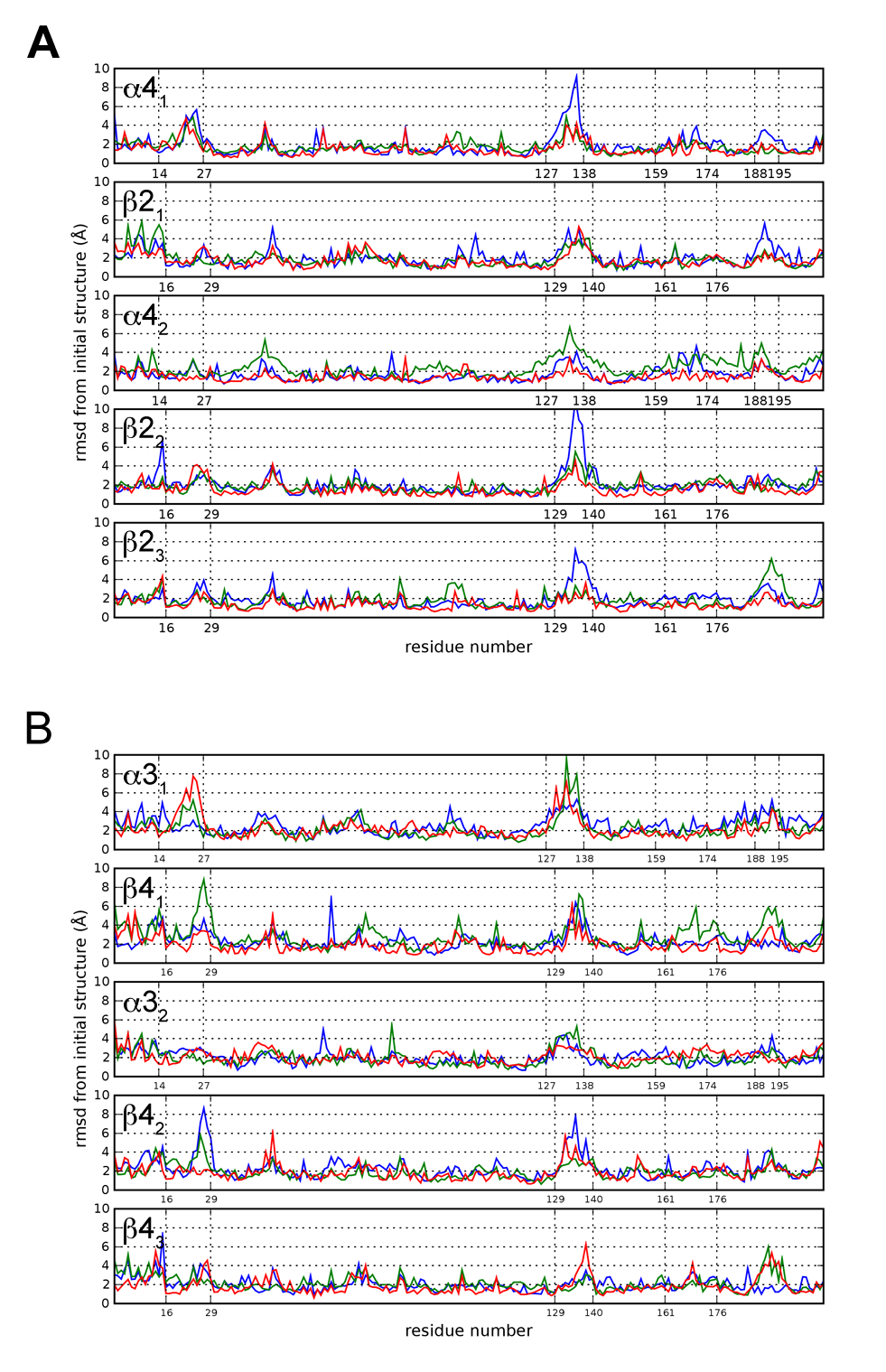

Supplement: Figure S4 — Average all-atom RMSDs for hα4β2 and hα3β4 nAChR ECD models in three different binding states. All-atom RMSD of each residue from the initial structure of a 5 ns MD simulation of three states: unbound (blue), bound to one epibatidine molecule at agonist binding site 1 (αx1/βx1 interface) (green), and bound to an epibatidine molecule at both agonist binding sites (red). Several loop regions are highlighted, including L1 (14-27), Cys-loop (127-138), F loop (159-174), and the α-subunit C loop (189-195). A. hα4β2 nAChR data B. hα3β4 nAChR data. (TIF) [file pone.0024949.s004.tif]

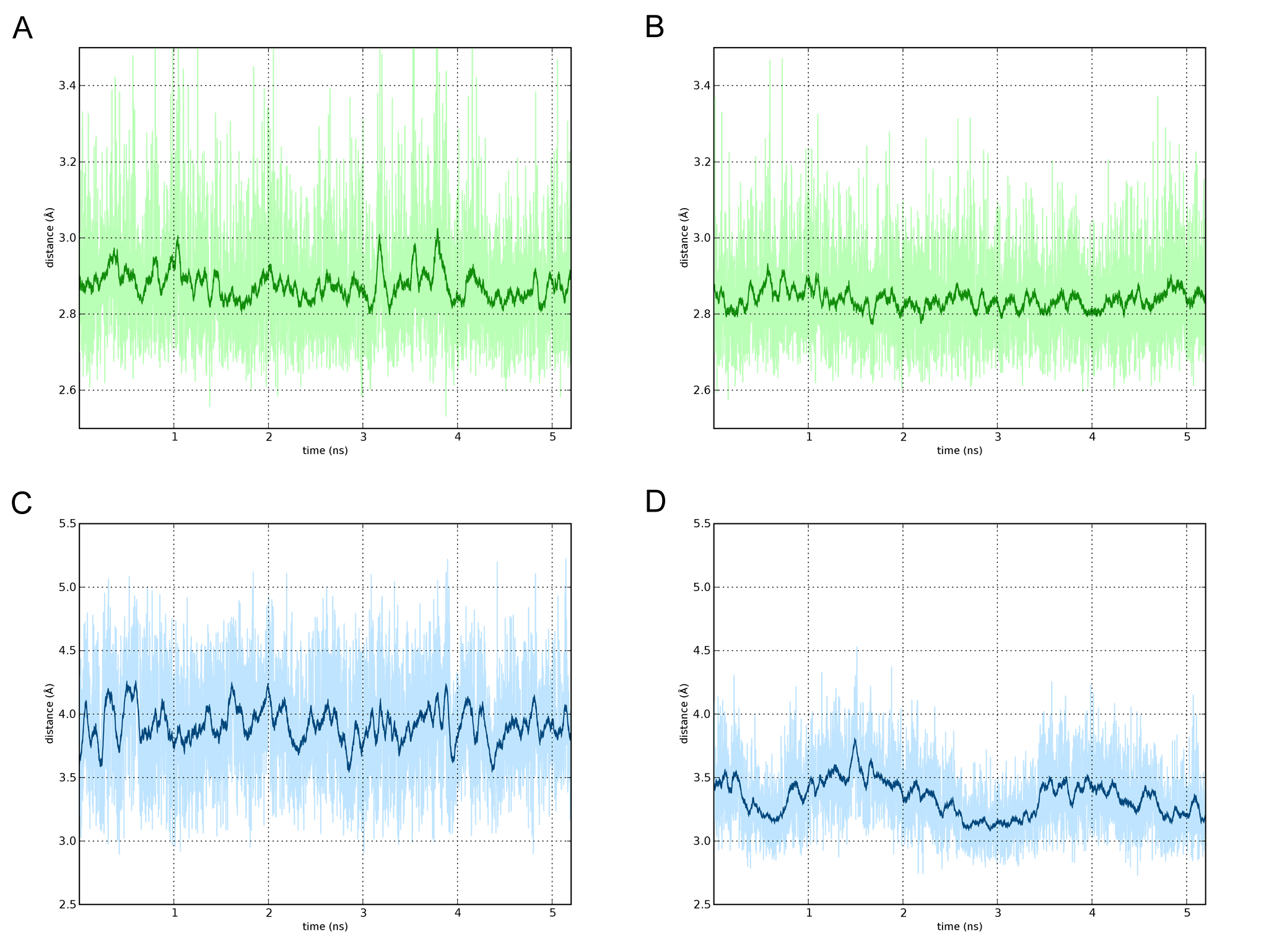

Supplement: Figure S5 — Measurement of epibatidine binding distances to hα4β2 and hα3β4 nAChR ECD binary complexes. Distance measurements quantifying epibatidine binding stability to agonist binding site 1 for hα4β2 (A, C) and hα3β4 (B, D) nAChR ECDs from 5 ns MD simulations. The distances between the positively charged nitrogen atom of epibatidine and both the backbone carbonyl oxygen atom of Trp148 (A, B) and the center of mass for the indole group of Trp148 (C, D) are measured. Picosecond interval data are plotted in the lighter color, while sliding average data with a window size of 50 data points are plotted in the darker color. (TIF) [file pone.0024949.s005.tif]

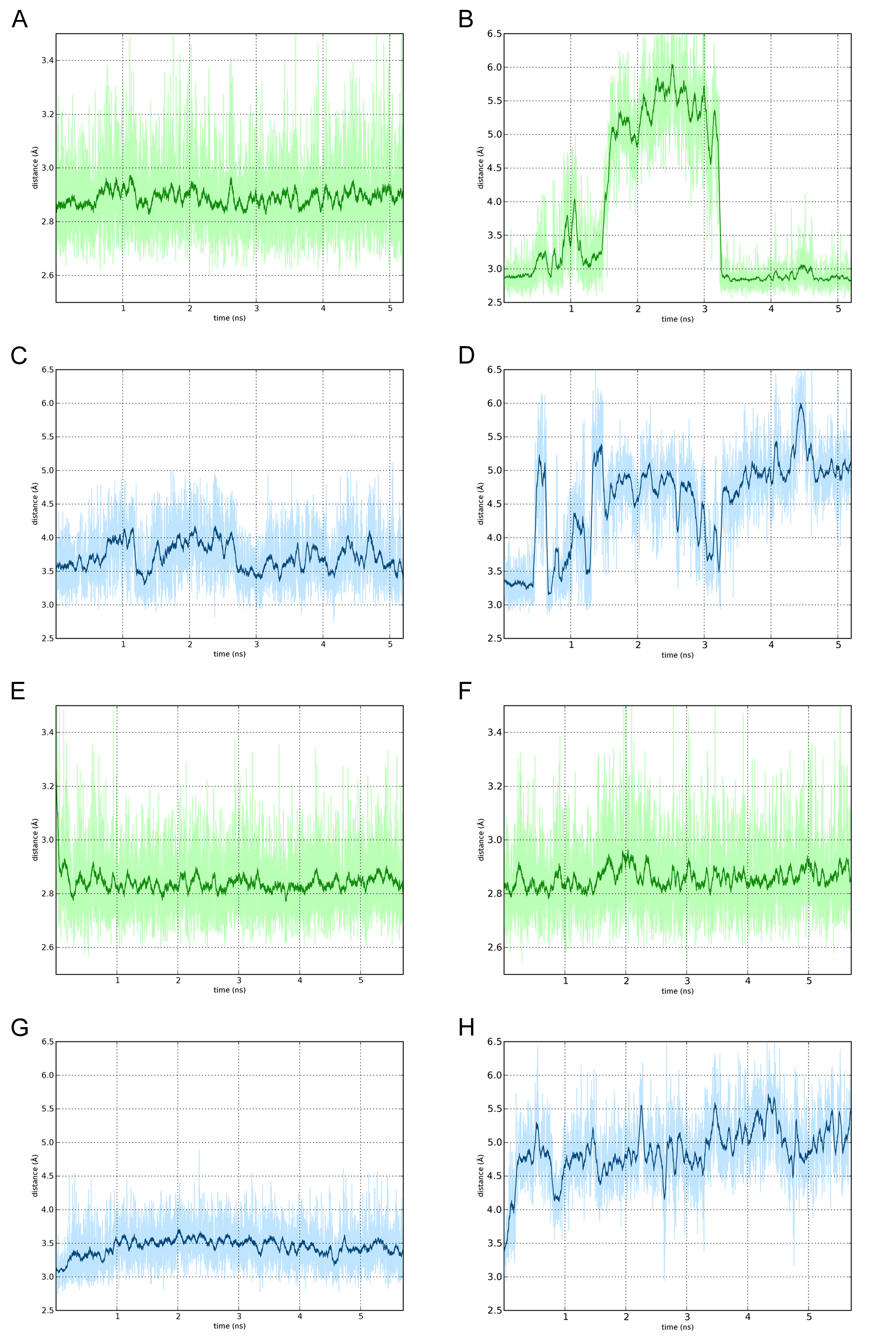

Supplement: Figure S6 — Measurement of epibatidine binding distances to hα4β2 and hα3β4 nAChR ECD ternary complexes. Distance measurements quantifying epibatidine binding stability to agonist binding site 1 (A, C, E, G) and binding site 2 (B, D, F, H) for hα4β2 (A, B, C, D) and hα3β4 (E, F, G, H) nAChR ECDs from 5 ns MD simulations. The distances between the positively charged nitrogen atom of epibatidine and both the backbone carbonyl oxygen atom of Trp148 (A, B, E, F) and the center of mass for the indole group of Trp148 (C, D, G, H) are measured. Picosecond interval data are plotted in the lighter color, while sliding average data with a window size of 50 data points are plotted in the darker color. (TIF) [file pone.0024949.s006.tif]

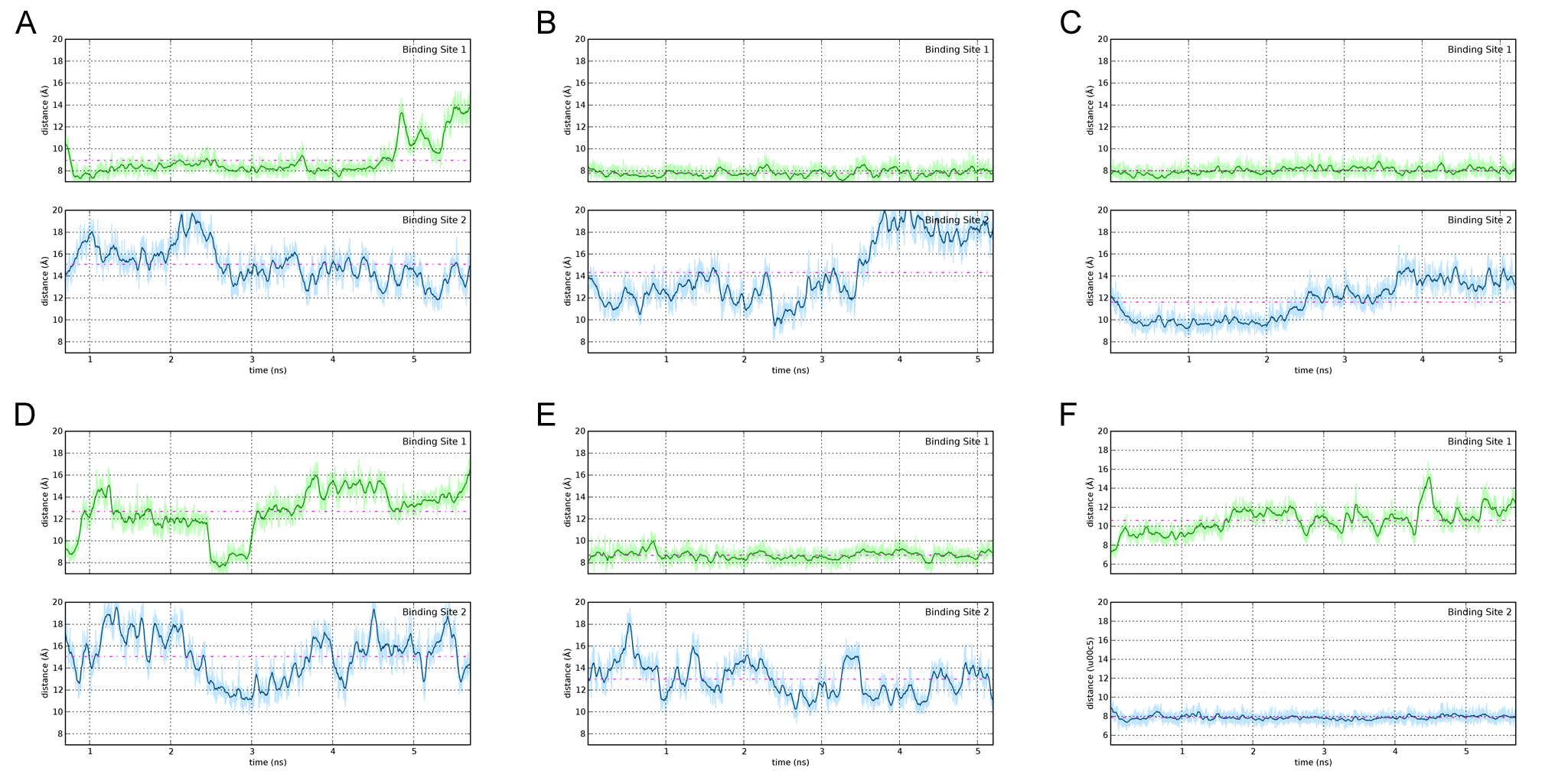

Supplement: Figure S7 — Measurements of C loop closure for hα4β2 and hα3β4 nAChR ECDs bound to epibatidine. Distance data that quantifies C loop dynamics upon agonist binding to the human α4β2 nAChR extracellular domains (A, B, C) and human α3β4 nAChR extracellular domains (D, E, F). The distance between Cα atoms of C191 on the C loop of α subunits on the (+) side of the binding interface and residue 58 on the β2 strand of β subunits on the (-) side of the interface is measure for unbound states (A, D), binary complexes (B, E), and ternary complexes (C, F). Distances are given at ps intervals (light-colored plots) and are also represented as sliding averages (dark-colored plots) with a window size of 50 data points. The magenta dashed lines are the average values for each plot. (TIF) [file pone.0024949.s007.tif]

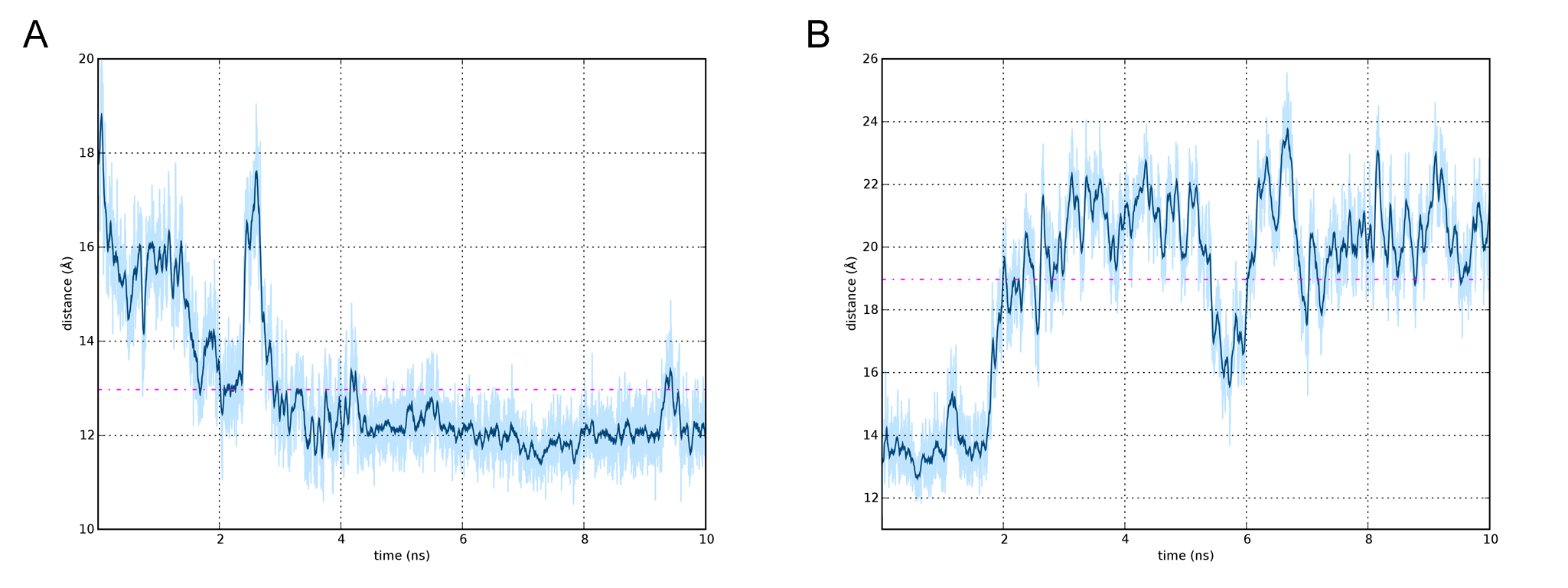

Supplement: Figure S8 — Measurements of C loop closure for hα4β2 and hα3β4 nAChR ECDs bound to both epibatidine and KAB-18. The distance between Cα atoms of C191 on the C loop of α subunits on the (+) side of the binding interface and residue 58 on the β2 strand of β subunits on the (-) side of the interface is measure for the hα4β2 (A) and hα3β4 nAChR ECDs bound to both epibatinde and KAB-18 at binding site 2. Distances are given at ps intervals (light-colored plots) and are also represented as sliding averages (dark-colored plots) with a window size of 50 data points. The magenta dashed lines are the average values for each plot. (TIF) [file pone.0024949.s008.tif]

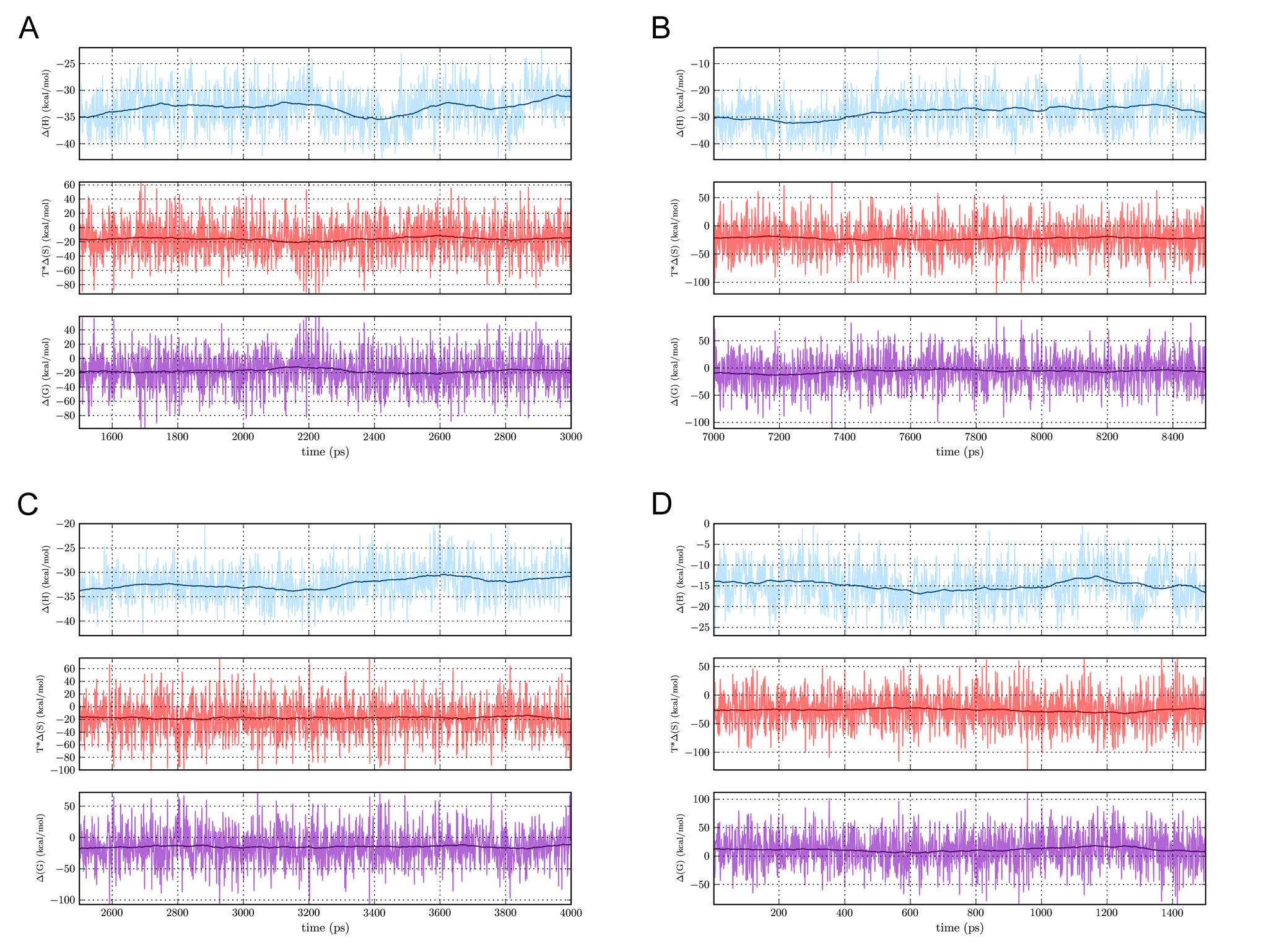

Supplement: Figure S9 — MMPB-SA binding energy calculations for epibatidine and KAB-18 binding. Binding energy components for epibatidine binding alone (A, C) and KAB-18 binding in the presence of epibatidine (B, D) to both hα4β2 (A, B) and hα3β4 (C, D) nAChR ECDs. Data is plotted at ps intervals (light trace) in addition to a sliding average trace (dark) with a window size of 200 data points. (TIF) [file pone.0024949.s009.tif]

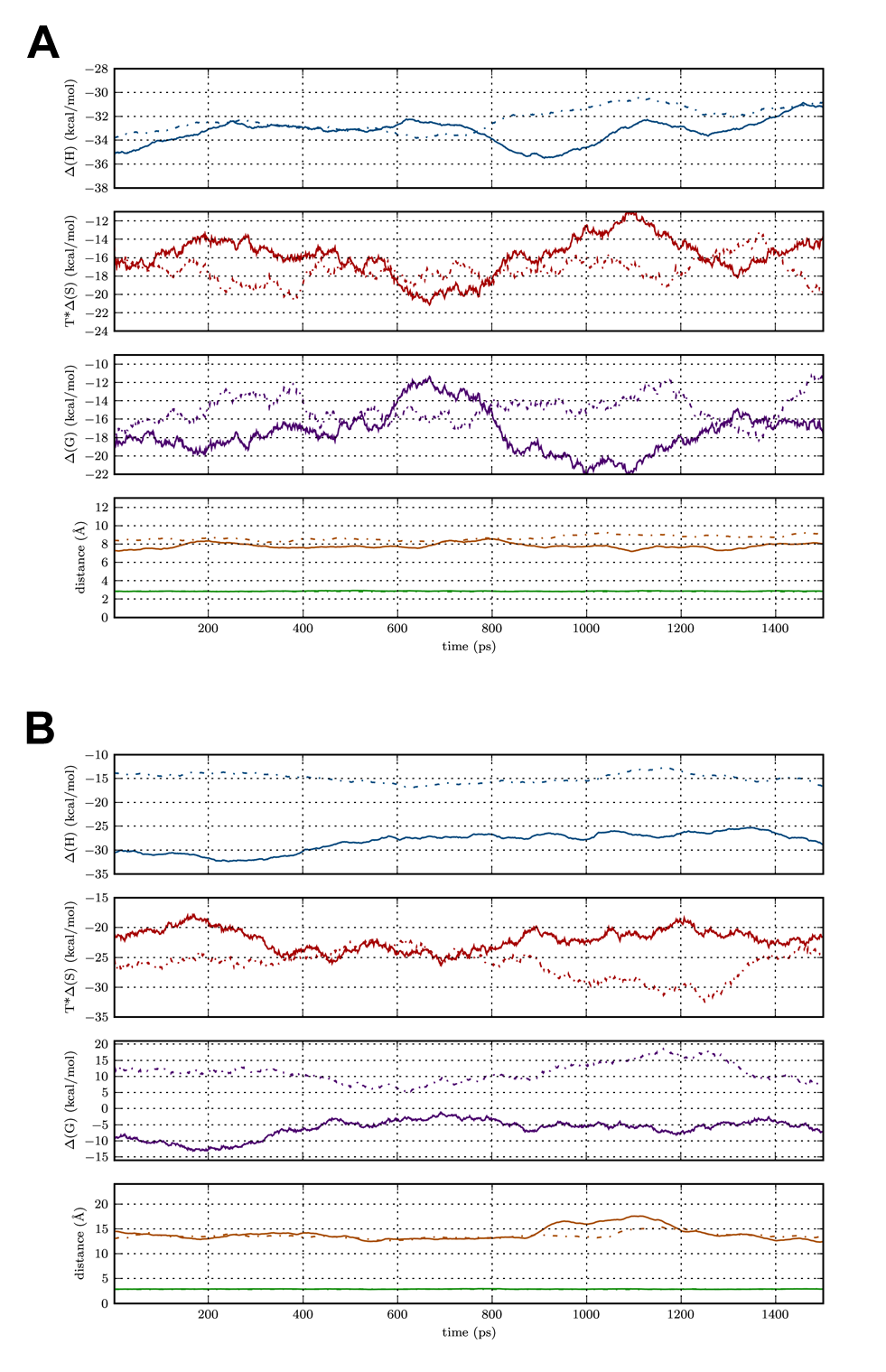

Supplement: Figure S10 — MMPB-SA binding energy calculations and dynamics analysis for ligand binding to hα4β2 and hα3β4 nAChRs. A. Epibatidine binding B. KAB-18 binding in the presence of epibatidine. The top three plots in each figure contain binding free energy data computed with the MM-PBSA protocol in Amber: ΔH (blue), TΔS (red), ΔG (purple). The bottom plot in each figure is distance data extracted from the MD simulations over the sampling period: distance between positively charged nitrogen atom of epibatidine and the backbone oxygen atom of Trp148 (green), and the Cα-Cα between α191 and β58 (yellow). The solid trace represents data for ligands bound to the hα4β2 nAChR extracellular domain model, while the dashed trace represents the hα3β4 nAChR data. All data is presented as sliding-window averages with a window size of 200 data points. (TIF) [file pone.0024949.s010.tif]

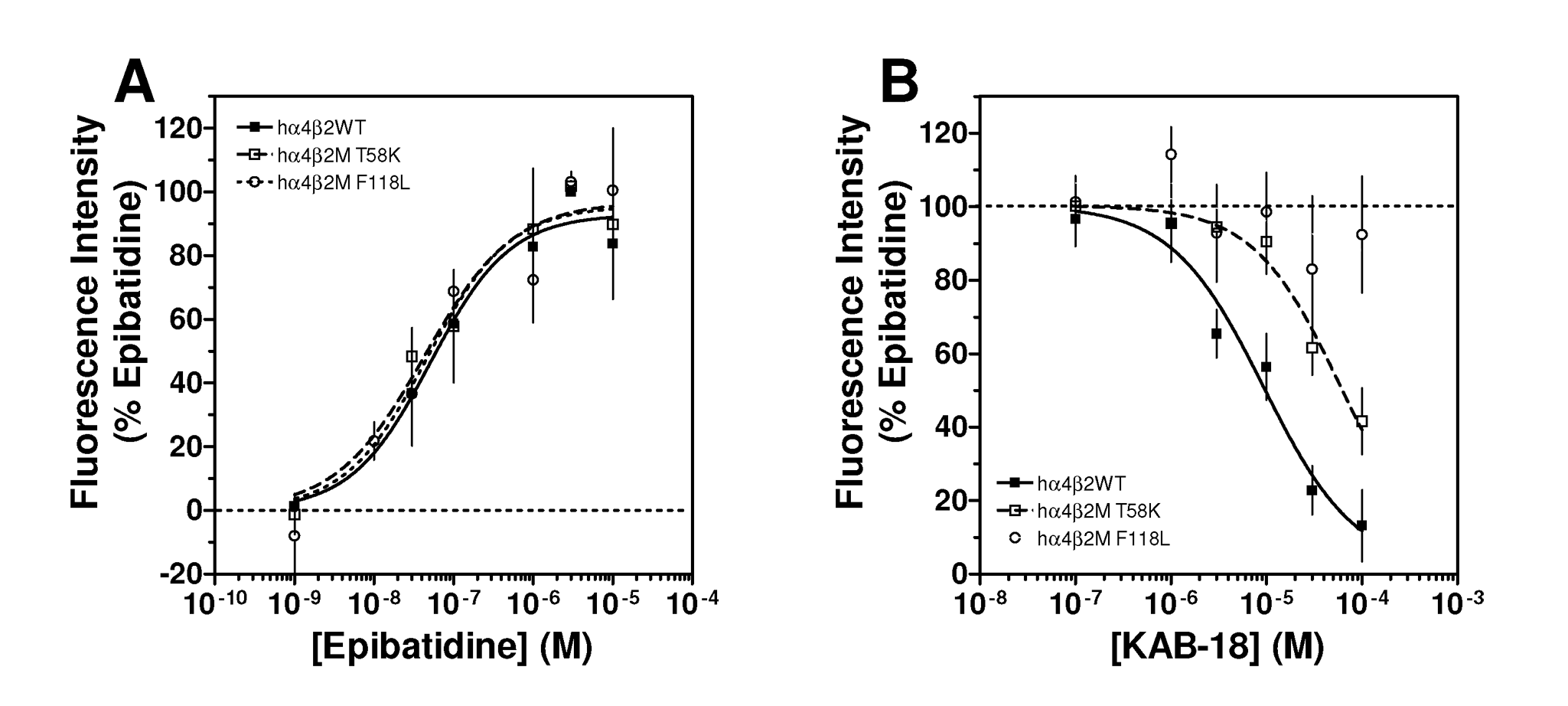

Supplement: Figure S11 — Dose-response curves for epibatidine and KAB-18 on wild-type and mutant hα4β2 nAChRs. A. Functional response for epibatidine binding to hα4β2WT (wild-type) and hα4β2M T58K/F118L mutant nAChRs. B. Functional response of KAB-18 on wild-type and mutant nAChRs. Data are expressed as a percentage of control responses using 3 µM epibatidine. Values represent means ± SEMs (n = 5 – 7). (TIF) [file pone.0024949.s011.tif]
