# Supplementary material for: Identification of a Negative Allosteric Site on Human α4β2 and α3β4 Neuronal Nicotinic Acetylcholine Receptors
Source: PLoS One. 2011 Sep 15;6(9):e24949. doi: 10.1371/journal.pone.0024949 (PMC3174232; doi:10.1371/journal.pone.0024949)
Supplement: Table S1 — Sequence identity between template and target sequences. (DOC) [file pone.0024949.s012.doc]

|  | L. stagnalis | A. californica | B. truncatus | *α1 ECD* | *hα3 ECD* | *rα3 ECD* | *hα4 ECD* | *hβ2 ECD* | *hβ4 ECD* | **rβ4 *ECD*** |
| --- | --- | --- | --- | --- | --- | --- | --- | --- | --- | --- |
| *L. stagnalis* | 100 |  |  |  |  |  |  |  |  |  |
| *A. californica* | 35.4 | 100 |  |  |  |  |  |  |  |  |
| *B. truncatus* | 47.1 | 36.5 | 100 |  |  |  |  |  |  |  |
| α1 ECD | 22.7 | 24.9 | 22.4 | 100 |  |  |  |  |  |  |
| hα3 ECD | 25.8 | 29.1 | 24.3 | 51.4 | 100 |  |  |  |  |  |
| rα3 ECD | 25.2 | 28.0 | 24.3 | 52.4 | 94.7 | 100 |  |  |  |  |
| hα4 ECD | 26.4 | 29.6 | 23.7 | 52.9 | 60.6 | 61.1 | 100 |  |  |  |
| hβ2 ECD | 24.5 | 26.1 | 27.8 | 44.9 | 49.5 | 51.0 | 54.0 | 100 |  |  |
| hβ4 ECD | 24.5 | 23.9 | 21.5 | 40.9 | 49.0 | 49.5 | 52.0 | 69.6 | 100 |  |
| **rβ4 ECD** | 23.9 | 22.2 | 21.5 | 41.4 | 48.0 | 61.5 | 50.0 | 68.6 | 92.8 | 100 |

Sequence identity between template sequences (AChBP of *Lymnaea stagnalis*, *Aplysia californica*, and *Bulinus truncatus* and the mouse α1 ECD) and the target sequences (the ECD of rat α3 and β4 subunits and human α3, α4, β2, and β4 subunits) in the aligned regions.
