# Supplementary material for: Identification of a Negative Allosteric Site on Human α4β2 and α3β4 Neuronal Nicotinic Acetylcholine Receptors
Source: PLoS One. 2011 Sep 15;6(9):e24949. doi: 10.1371/journal.pone.0024949 (PMC3174232; doi:10.1371/journal.pone.0024949)
Supplement: Table S2 — Sequence similarity between template and target sequences. (DOC) [file pone.0024949.s013.doc]

|  | L. stagnalis | A. californica | B. truncatus | *α1 ECD* | *hα3 ECD* | *rα3 ECD* | *hα4 ECD* | *hβ2 ECD* | *hβ4 ECD* | **rβ4** *ECD* |
| --- | --- | --- | --- | --- | --- | --- | --- | --- | --- | --- |
| *L. stagnalis* | 100 |  |  |  |  |  |  |  |  |  |
| *A. californica* | 59.0 | 100 |  |  |  |  |  |  |  |  |
| *B. truncatus* | 68.6 | 60.5 | 100 |  |  |  |  |  |  |  |
| α1 ECD | 44.2 | 46.6 | 44.7 | 100 |  |  |  |  |  |  |
| hα3 ECD | 48.5 | 45.0 | 48.7 | 69.7 | 100 |  |  |  |  |  |
| rα3 ECD | 48.5 | 43.9 | 49.3 | 68.8 | 96.2 | 100 |  |  |  |  |
| hα4 ECD | 42.9 | 42.9 | 44.7 | 66.8 | 76.4 | 76.4 | 100 |  |  |  |
| hβ2 ECD | 45.8 | 43.9 | 47.2 | 65.7 | 68.7 | 70.7 | 69.2 | 100 |  |  |
| hβ4 ECD | 48.4 | 43.9 | 49.3 | 63.6 | 68.7 | 69.7 | 68.2 | 85.5 | 100 |  |
| **rβ4 ECD** | 48.4 | 43.3 | 49.3 | 64.1 | 67.7 | 68.7 | 67.7 | 85.0 | 97.6 | 100 |

Sequence similarity between template sequences (AChBP of *Lymnaea stagnalis*, *Aplysia californica*, and *Bulinus truncatus* and the mouse α1 ECD) and the target sequences (the ECD of rat α3 and β4 subunits and human α3, α4, β2, and β4 subunits) in the aligned regions.
