# Supplementary material for: Identification of a Negative Allosteric Site on Human α4β2 and α3β4 Neuronal Nicotinic Acetylcholine Receptors
Source: PLoS One. 2011 Sep 15;6(9):e24949. doi: 10.1371/journal.pone.0024949 (PMC3174232; doi:10.1371/journal.pone.0024949)
Supplement: Table S3 — Average RMSDs for backbone atoms of α4β2 and α3β4 nAChR ECD models from MD simulations in three states. (DOC) [file pone.0024949.s014.doc]

|  |  |  | apo | binary | ternary |  |  | apo | binary | ternary |
| --- | --- | --- | --- | --- | --- | --- | --- | --- | --- | --- |
| α4β2  average RMSD  (Å) | α4_1_ | all residues | 1.91 | 1.62 | 1.40 | α3β4  average RMSD  (Å) | α3_1_ | 2.44 | 2.16 | 2.18 |
|  |  | C loop | 2.60 | 1.07 | 1.30 |  |  | 3.48 | 2.87 | 2.26 |
|  |  | F loop | 1.84 | 1.64 | 1.14 |  |  | 2.08 | 1.55 | 1.36 |
|  |  | A loop | 1.29 | 1.42 | 1.33 |  |  | 2.10 | 1.51 | 1.50 |
|  |  | Loop 1 | 3.08 | 2.55 | 2.57 |  |  | 2.42 | 2.77 | 4.44 |
|  |  | Cys loop | 4.35 | 2.15 | 2.04 |  |  | 4.12 | 4.06 | 3.84 |
|  |  | B loop | 1.49 | 0.92 | 0.95 |  |  | 1.85 | 2.31 | 1.44 |
|  | β2_1_ | all residues | 1.82 | 1.77 | 1.66 |  | β4_1_ | 2.15 | 2.79 | 1.75 |
|  |  | C loop | 3.65 | 2.10 | 1.92 |  |  | 1.87 | 4.70 | 2.80 |
|  |  | F loop | 1.60 | 1.59 | 1.33 |  |  | 1.86 | 3.23 | 1.56 |
|  |  | A loop | 1.47 | 1.37 | 0.95 |  |  | 1.38 | 2.36 | 1.95 |
|  |  | Loop 1 | 1.57 | 1.66 | 1.92 |  |  | 2.98 | 4.56 | 2.25 |
|  |  | Cys loop | 3.00 | 2.49 | 2.73 |  |  | 2.99 | 3.39 | 2.08 |
|  |  | B loop | 1.22 | 1.42 | 1.13 |  |  | 1.85 | 1.74 | 1.30 |
|  | α4_2_ | all residues | 1.55 | 2.38 | 1.27 |  | α3_2_ | 1.90 | 1.83 | 1.96 |
|  |  | C loop | 2.27 | 2.98 | 2.19 |  |  | 2.61 | 1.90 | 1.54 |
|  |  | F loop | 2.22 | 2.54 | 1.15 |  |  | 1.57 | 1.63 | 2.25 |
|  |  | A loop | 1.34 | 2.41 | 1.26 |  |  | 1.38 | 0.99 | 2.31 |
|  |  | Loop 1 | 1.64 | 1.75 | 1.21 |  |  | 2.61 | 1.97 | 1.94 |
|  |  | Cys loop | 1.60 | 1.59 | 1.33 |  |  | 1.86 | 3.23 | 1.56 |
|  |  | B loop | 1.47 | 1.37 | 0.95 |  |  | 1.38 | 2.36 | 1.95 |
|  | β2_2_ | all residues | 1.57 | 1.66 | 1.92 |  | β4_2_ | 2.98 | 4.56 | 2.25 |
|  |  | C loop | 3.00 | 2.49 | 2.73 |  |  | 2.99 | 3.39 | 2.08 |
|  |  | F loop | 1.22 | 1.42 | 1.13 |  |  | 1.85 | 1.74 | 1.30 |
|  |  | A loop | 1.55 | 2.38 | 1.27 |  |  | 1.90 | 1.83 | 1.96 |
|  |  | Loop 1 | 2.27 | 2.98 | 2.19 |  |  | 2.61 | 1.90 | 1.54 |
|  |  | Cys loop | 2.22 | 2.54 | 1.15 |  |  | 1.57 | 1.63 | 2.24 |
|  |  | B loop | 1.34 | 2.41 | 1.26 |  |  | 1.38 | 0.99 | 2.31 |
|  | β2_2_ | all residues | 1.64 | 1.75 | 1.21 |  | β4_2_ | 2.61 | 1.97 | 1.94 |
|  |  | C loop | 1.60 | 1.59 | 1.33 |  |  | 1.86 | 3.23 | 1.56 |
|  |  | F loop | 1.47 | 1.37 | 0.95 |  |  | 1.38 | 2.36 | 1.95 |
|  |  | A loop | 1.57 | 1.66 | 1.92 |  |  | 2.98 | 4.56 | 2.25 |
|  |  | Loop 1 | 3.00 | 2.49 | 2.73 |  |  | 2.99 | 3.39 | 2.08 |
|  |  | Cys loop | 1.22 | 1.42 | 1.13 |  |  | 1.85 | 1.74 | 1.30 |
|  |  | B loop | 1.55 | 2.38 | 1.27 |  |  | 1.90 | 1.83 | 1.96 |

Average RMSDs for backbone atoms of α4β2 and α3β4 nAChR ECD models from MD simulations in three states: unbound, binary complex (one bound epibatidine molecule at αx_1_/βx_1_ interface, agonist binding site 1 in Figure 1) and ternary complex (an epibatidine molecule bound to each α/β interface, agonist binding sites 1 and 2 in Figure 1). MD snapshots were collected at 1 ps intervals from 5 ns long trajectories, and all RMSD values are in reference to the initial structure of each trajectory.
