# Supplementary material for: Identification of a Negative Allosteric Site on Human α4β2 and α3β4 Neuronal Nicotinic Acetylcholine Receptors
Source: PLoS One. 2011 Sep 15;6(9):e24949. doi: 10.1371/journal.pone.0024949 (PMC3174232; doi:10.1371/journal.pone.0024949)
Supplement: Table S4 — Survey of C loop closure for AChBP X-ray structures. (DOC) [file pone.0024949.s015.doc]

| PDB ID | Structure resolution (Å) | Compound name | Measurement of C loop closure (Å)^a^ | Compound type |
| --- | --- | --- | --- | --- |
| 2WNL (1) | 2.70 | anabaseine | 7.72 | agonist |
| 2BYQ (2) | 3.40 | epibatidine | 7.80 | agonist |
| 1UW6 (3) | 2.20 | nicotine | 7.93 | agonist |
| 2BJ0 (4) | 2.00 | CXS | 8.10 | buffer |
| 1UV6 (3) | 2.50 | carbamylcholine | 8.16 | agonist |
| 2BYS (2) | 2.05 | lobeline | 8.19 | agonist |
| 2BR7 (5) | 3.00 | HEPES | 8.33 | buffer |
| 1I9B (6) | 2.70 | HEPES | 9.30 | buffer |
| 1UX2 (3) | 2.20 | HEPES | 9.34 | buffer |
| 2WNJ (1) | 1.80 | DMXBA | 9.75 | partial agonist |
| 2WNC (1) | 2.20 | tropisetron | 10.13 | partial agonist |
| 2WN9 (1) | 1.75 | 4-OH-DMXBA | 12.30 | partial agonist |
| 2X00 (7) | 2.40 | gymnodimine A | 12.88 | antagonist |
| 2BYN (2) | 2.02 | PEG | 14.71 | buffer |
| 2BYR (2) | 2.45 | methyllycaconitine | 14.64 | antagonist |
| 2BG9 (8) | 4.00 | - | 15.36 | - |
| 2W8E (9) | 1.90 | - | 15.72 | - |
| 2WZY (7) | 2.51 | 13-desmethyl spirolidine | 16.05 | antagonist |
| 1YI5 (10) | 4.20 | cobratoxin | 17.50 | peptidic antagonist |
| 2BR8 (5) | 2.40 | α-conotoxin PNIA | 18.76 | peptidic antagonist |
| 2C9T (11) | 2.25 | α-conotoxin IMI | 19.13 | peptidic antagonist |
| 2BYP (2) | 2.07 | α-conotoxin IMI | 19.24 | peptidic antagonist |

^a^Average Cα-Cα distance between residues that correspond to C191 on the C loop of nAChR α subunit of the (+) of the binding interface and residue 58 on the β2 strand of β subunits of the (-) side of the interface

**SUPPORTING REFERENCES**

1. Hibbs, R. E., Sulzenbacher, G., Shi, J. X., Talley, T. T., Conrod, S., Kem, W. R., Taylor, P., Marchot, P., and Bourne, Y. (2009) *Embo Journal* **28**, 3040-3051

2. Hansen, S. B., Sulzenbacher, G., Huxford, T., Marchot, P., Taylor, P., and Bourne, Y. (2005) *EMBO J* **24**, 3635-3646

3. Celie, P. H., van Rossum-Fikkert, S. E., van Dijk, W. J., Brejc, K., Smit, A. B., and Sixma, T. K. (2004) *Neuron* **41**, 907-914

4. Celie, P. H., Klaassen, R. V., van Rossum-Fikkert, S. E., van Elk, R., van Nierop, P., Smit, A. B., and Sixma, T. K. (2005) *J Biol Chem* **280**, 26457-26466

5. Celie, P. H., Kasheverov, I. E., Mordvintsev, D. Y., Hogg, R. C., van Nierop, P., van Elk, R., van Rossum-Fikkert, S. E., Zhmak, M. N., Bertrand, D., Tsetlin, V., Sixma, T. K., and Smit, A. B. (2005) *Nat Struct Mol Biol* **12**, 582-588

6. Brejc, K., van Dijk, W. J., Klaassen, R. V., Schuurmans, M., van Der Oost, J., Smit, A. B., and Sixma, T. K. (2001) *Nature* **411**, 269-276

7. Bourne, Y., Radic, Z., Araoz, R., Talley, T. T., Benoit, E., Servent, D., Taylor, P., Molgo, J., and Marchot, P. *Proc Natl Acad Sci U S A* **107**, 6076-6081

8. Unwin, N. (2005) *J Mol Biol* **346**, 967-989

9. Ulens, C., Akdemir, A., Jongejan, A., van Elk, R., Bertrand, S., Perrakis, A., Leurs, R., Smit, A. B., Sixma, T. K., Bertrand, D., and de Esch, I. J. (2009) *J Med Chem* **52**, 2372-2383

10. Bourne, Y., Talley, T. T., Hansen, S. B., Taylor, P., and Marchot, P. (2005) *Embo Journal* **24**, 1512-1522

11. Ulens, C., Hogg, R. C., Celie, P. H., Bertrand, D., Tsetlin, V., Smit, A. B., and Sixma, T. K. (2006) *Proc Natl Acad Sci U S A* **103**, 3615-3620
